# Biosynthesis of JC-La_2_CoO_4_ magnetic nanoparticles explored in catalytic and SMMs properties

**DOI:** 10.1038/s41598-023-47852-9

**Published:** 2023-12-13

**Authors:** Nilesh Satpute, Mithun Kumar Ghosh, Aparna Kesharwani, Tanmay Kumar Ghorai

**Affiliations:** 1 Nanomaterials and Crystal Design Laboratory, Department of Chemistry, Indira Gandhi National Tribal University, Amarkantak, Madhya Pradesh 484887 India; 2 Department of Chemistry, Govt. College Hatta, Damoh, Madhya Pradesh 470775 India

**Keywords:** Chemistry, Materials science, Nanoscience and technology

## Abstract

We have reported the synthesis of JC-La_2_CoO_4_ magnetic nanoparticles from *Jatropha Curcas L.* leaf extract in aqueous medium and potential application study in catalytic & Single Molecule Magnets (SMMs). Several techniques were used to investigate the structural, morphological, and elemental composition, particle size, optical properties, catalytic and magnetic properties by XRD, FTIR, SEM, EDAX, XPS, UV–visible and squid magnetic measurement. It was found that the crystallite sizes and grain sizes of JC-La_2_CoO_4_ NPs were 11.3 ± 1 and 24.1 ± 1 nm respectively and surface morphology of the nanoparticles looks spherical shape with good surface area. The band gap of JC-La_2_CoO_4_ was found to be 4.95 eV indicates good semiconductor in nature. XPS studies shows that La and Co present in + 3 and + 2 oxidation state respectively and suggest the composition formula is La_2_CoO_4_ with satisfied all the valency of metal ions. The photocatalytic efficiency of La_2_CoO_4_ shows good result against methylene blue (MB) compared to other dyes like MO, NO, RhB in presence of sunlight with rate constant 56.73 × 10^–3^ min^-1^ and completely degraded within 115 mints. The importance of JC-La_2_CoO_4_ has magnetic properties with antiferromagnetic coupling and SMMs properties with nature.

## Introduction

Synthesis of bimetallic nanoparticles is very attraction to their vast application in industrial, medicinal, optical, electronic, magnetic, and catalyst properties^1–5^. Plant extraction mediated green synthesis provide cost effectiveness, simple, stable and non toxic. Plant extract is used as a reducing and stabilizing environment friendly reducing agent for synthesis of nanoparticles^1^. *Jatropha curcas L.* (JC) is the family of Euphorbiaceae and used as herbal medicine and leaf extract used as anti-malaria medicine^6,7^. Green synthesizes 3d and 4f. novel metals in the field of magnetic nanoparticles (MNPs) has always been a challenging task and recently researchers have attracted enormous attention in this area because these magnetic nanoparticles show distinguishing properties and are significantly different from bulk materials for many new potential applications^8–10^. Lanthanum oxide is an optically active sesquioxide among all other rare earth metal oxides^11,12^. Simultaneously, among the transition metals cobalt metal has multiple properties like being a semiconducting material, magnetic, catalytic and considerable attention^4,13–15^. Dyes are chemical pollutants that are the root cause of water resource contamination. Nowadays, dyes are being used in the textile, pigment, photographic, leather, cosmetics, and paper industries to determine the attractiveness of consumers. A bulk of contaminated industrial effluents are discharged into bodies of water without their possible treatment^16,17^. The majority of dye industry waste water is contains diverse natural azo colours, which are toxic and these colours are exceptionally harmful in nature and unsafe to sea animals and in addition to the human being^18–20^. Azo dyes, for example, naphthol orange, methyl orange, rhodamine B, conga red were the principal materials in the dye industry.^21,22^ Simultaneously, methylene blue is also a cationic thiazine dye known as methylthioninium chloride and used in the textile, paint industries and has an effect on the central nervous system^23^.

At present, the green method is an advanced methodology for the removal of toxic substances and the detoxification of dyes using UV radiation, toxic stabilizer, surfactants, and microbial degradation^24–26^. Biologically synthesising bimetallic nanoparticles for catalytic reduction of organic dyes received the researcher’s attention due to the high potential of degradation of dyes. However, methods for the synthesis of suitable photocatalyst nanoparticles using plant extract are prevalent but the application of nanoparticles in the treatment of different dye effluents is limited^27^. A photocatalyst is one of the most effective techniques for the degradation of organic dye without producing any toxic byproduct at the end of the process.

Therefore, our objective is to remove the dye pollutants from waste water by using synthesized magnetic nanoparticles from the green technique. However, there are several examples of green synthesize of single metal nanoparticles like CeO_2_, AgNPs, AuNPs, PtNPs, carbon-lignin/ZnO, Co_3_O_4_, Fe_3_O_4_ nanoparticles that can be used as excellent corrosion inhibitor, indoor air pollutant degradants, photocatalysts, magnetic nanoparticle for wastewater treatment, anticancer, antimicrobial and biomedical applications^12,16,28–38^. Bimetallic magnetic nanoparticles have been reported for catalytic applications, sensors, and the biomedical field and are mainly synthesised from organic precursors or solvents^39–42^. But here we are very much emphasizing the establishment of the biosynthesis of bimetallic magnetic nanoparticles (i.e. La and Co-based metal ions) from plant extract and their study in dual applications like catalytic and single-molecule magnets, which is very rare. Advantages of biosynthesis do not require adding capping agents for stabilize the compounds because bio extract (*Jatropha Curcas*) itself is used as an oxidizing and reducing agent and stabilizes the magnetic nanoparticles. Single-molecules magnets are very interesting, mainly used in data storage, exchange bias materials, etc. and mostly obtained from organic precursors^43–45^. Accordingly, the present research work deals with the green synthesis of the magnetic nanoparticle JC-La_2_CoO_4_ using *Jatropha curcas L.* (JC) leaf extract, lanthanum and cobalt. JC leaf extract contains the phytochemicals i.e. flavonoids, alkaloids, terpenoids, phenolic acids, amines, tannins, saponins and may responsible for the reduction of La^+3^ to La and Co^+2^ to Co NPs^26,46^. The spinel structure of JC-La_2_CoO_4_ nanoparticles has been established from XRD, FTIR and XPS measurements and extensively studied in optical, catalytic (degradation of methylene blue) and magnetic properties.

## Experimental methods

### Materials and methods

Lanthanum nitrate [La (NO_3_)_3_.6H_2_O] (Alfa Aesar 99.9%), Cobalt nitrate [Co (NO_3_)_3_.6H_2_O] Merck (99.0%), Milli-Q water, All the chemicals and reagent are purchased, and used without purification.

### Plant materials

**Figure 1:**
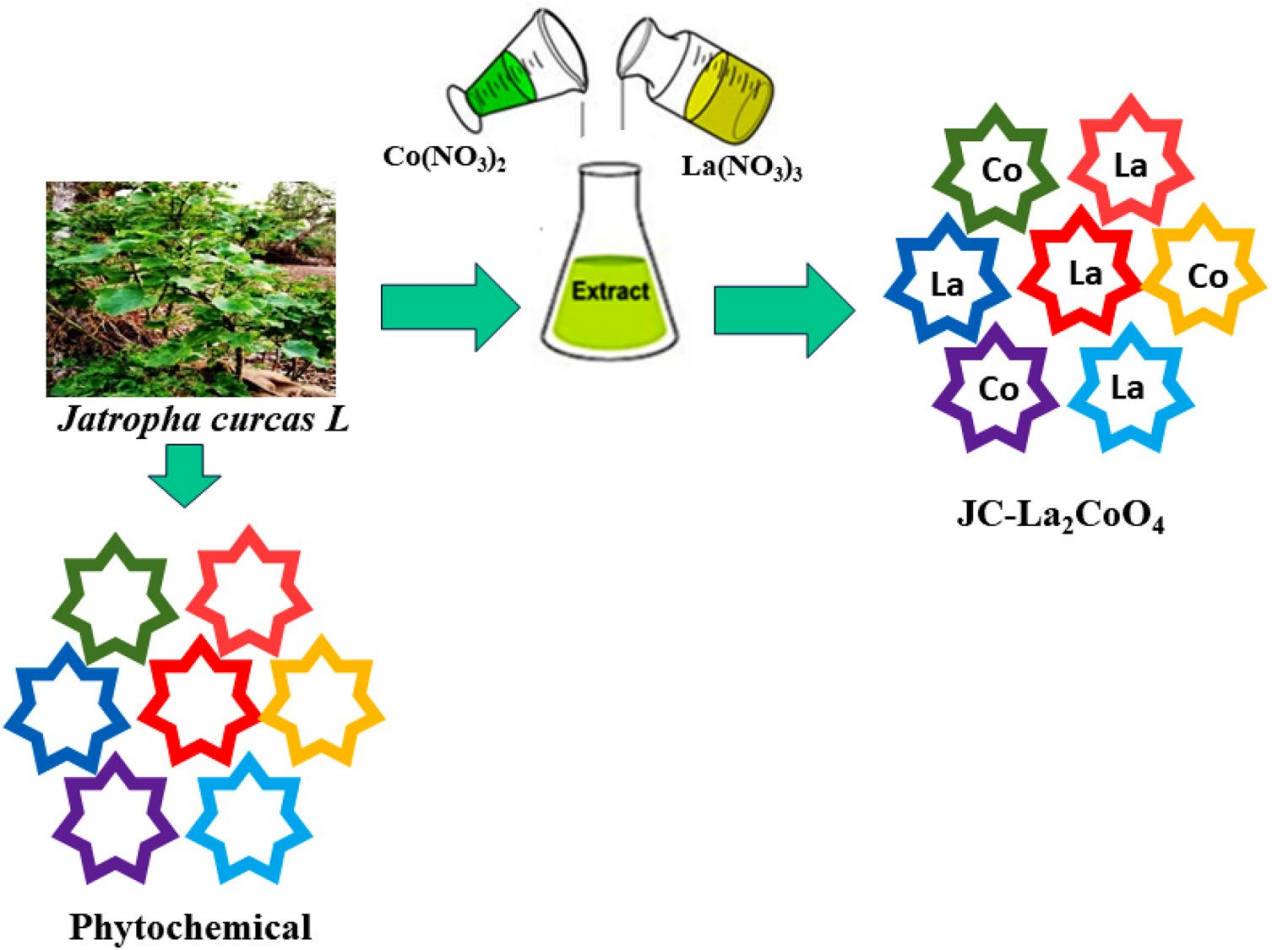
Green Synthesis of JC-La_2_CoO_4_ NPs.

Jatropha curcas L. (family: Euphorbiaceae) is a perennial shrub widely cultivated in the Amarkantak region as a living fence (hedge) in the fields and human settlements. The IUCN status of the Plant is 'Least concern'. The authentication of the plant species was identified by a plant taxonomist (Dr. Ravindra Shukla) and its physical specimen (IGNTU/DoB/2023/Eup/JC/06) was lodged in the herbarium of the Department of Botany, Indira Gandhi National Tribal University, Amarkantak as per national, and international guidelines and legislation. The wild plant *Jatropha curcas* (JC) leaves were collected by Ghorai Research Group (N. Satpute, A. Kesharwani and M. K. Ghosh) from the Podki near the Indira Gandhi National Tribal University, Amarkantak, Madhya Pradesh, India in the month of April 2023 (Fig. 1). The research work of JC was completed in the Department of Chemistry, Nanomaterials & Crystal Design Laboratory, Indira Gandhi National Tribal University, Madhya Pradesh, Amarkantak, India.

### Preparation of the JC plant extract

An extract of *Jatropha curcas* leaf was used for the green synthesis of JC-La_2_CoO_4_. The leaves of *Jatropha curcas* were washed with running tap water to remove debris and other contaminated particles, followed by double distilled water (DDW) twice, and air dried. Collect the fresh leaves and remove the debris and dust by running tap water. Wash the leaves with purified water and then Milli-Q water, and then cut them into small pieces before being dried. The 15 g of dried leaves are finally cut and kept in a beaker immersed in 150 mL of Milli-Q water and heated to boiling for up to 25 min. Cooled the solution and filtered with Whatman (41) filter paper and used for the synthesis of JC-La_2_CoO_4_ or stored for further use at 4 °C.

### Phytochemical analysis

To identifying the major phytochemicals in the sale followed by the standard protocol for qualitative phytochemicals analysis^13,14^, shown in the Table 1.

**Table 1:** Phytochemical analysis of *Jatropha curcas* leaf.

S. No	Phytochemical Test	Result
1	Flavonoids	–
2	Tannins	+
3	Phlobatannins	–
4	Terpenoids	–
5	Steroids	–
6	Saponins	+
7	Glycosides	–
8	Phenol	+
9	Alkaloids	+
10	Phytosterols	–
11	Anthocyanin	–
12	Anthraquinone	–

### Bio-synthesis of JC- La_2_CoO_4_ NPs

At first, La(NO_3_)_3_ (0.389 g, 10 mM) was taken in a 200 ml beaker and dissolved in 90 mL of DDW. After that, 10 mL of JC leave extract was added to the La(NO_3_)_3_ solution with a 9:1 ratio, and the mixture was kept at room temperature and stirred for about 15 min. In another beaker, a Co(NO_3_)_2_ (0.262 g, 10 mM) solution was prepared by dissolving in 90 mL of DDW. After that, cobalt nitrate solution was added to the mixture of JC-La- extract and stirred constantly for about 2 h and then the mixture was kept in a hot air oven at 35 °C for overnight. A greenish yellow viscous solution of JC-La_2_CoO_4_ NPs has been obtained as a suspended particle and confirmed by UV- visible spectroscopy measurement. The pH of the reaction mixture is 4.15 during the preparation of JC-La_2_CoO_4_ NPs. Finally, the resultant solution was centrifuged at 11,000 rpm for 15 min at room temperature and JC-La_2_CoO_4_ NPs were precipitated at the bottom of the centrifuge tube, filtered and washed with purified water, dried in an oven at 80 °C for 2 h and collected as brown JC-La_2_CoO_4_ for further characterization. A phytochemical test of JC was performed and it may act as reducing as well as capping and stabilizing agents for the green synthesis of JC-Co_2_LaO_4_ NPs. The schematic representation of JC-Co_2_LaO_4_ is shown in the Fig. 1.

### Characterization techniques

JC-La_2_CoO_4_ NPs formation, optical property, and photocatalytic activity have been characterized by the use of the UV visible spectrophotometer (Shimadzu UV-1800). The crystal structure of JC-La_2_CoO_4_ NPs was measured by Powder X-ray diffraction (XRD) at room temperature by using Xʹ Pert3 Panalytical, equipped with Cu Kα (1.54060 Å) as the incident radiation. Scherer equation was used for calculation of crystallite size. The Scherer equation was D = Kλ/*β*cosӨ, K = 0.9, D = Crystal size (Å), λ = Wavelength of Cu-Kα radiation, and β = Corrected half width of the diffraction peak. Nicolet iS5 (Thermo Scientific) was used for FT-IR analysis of samples at room temperature. The surface morphology and elemental composition of the fine NPs were analysed by Scanning Electronic Microscopy (SEM) and EDAX (SEM-EDAX: JEOL 6390LA/ OXFORD XMX N). Oxidation state of metals with presence of elemental % of nanomaterial measured by X-ray Photoelectron Spectroscopy (Thermo Fisher Scientific: Escalab Xi+). Magnetic Study of prepared sample DC and AC magnetic susceptibility were carried out on Superconducting Quantum Interference Device Magnetometry. Quantum Design MPMS-XL SQUID magnetometer (IISER Bhopal) equipped with a 7-T magnet and operating in the 1.8 to 300-K range was used on vacuum dried solids to collect variable-temperature dc and ac magnetic susceptibility data.

### Photocatalytic experiment

Photocatalytic experiments were conducted using JC-La_2_CoO_4_ NPs, under aqueous solution of naphthol orange (NO), methylene blue (MB), rhodamine B (Rh B) and methyl orange (MO) in presence of sunlight. The reactions were performed by adding synthesized nanoparticles (0.1 g) into each set of a 20 mL dye, which is standardized. In each set of reaction solutions were measured by UV–VIS spectrophotometer (UV-1800, Shimadzu) after 10 min intervals. The maximum absorbance of MB is at 662 nm.

## Results and discussion

### Green synthesis of JC-La_2_CoO_4_ NPs

**Figure 2:**
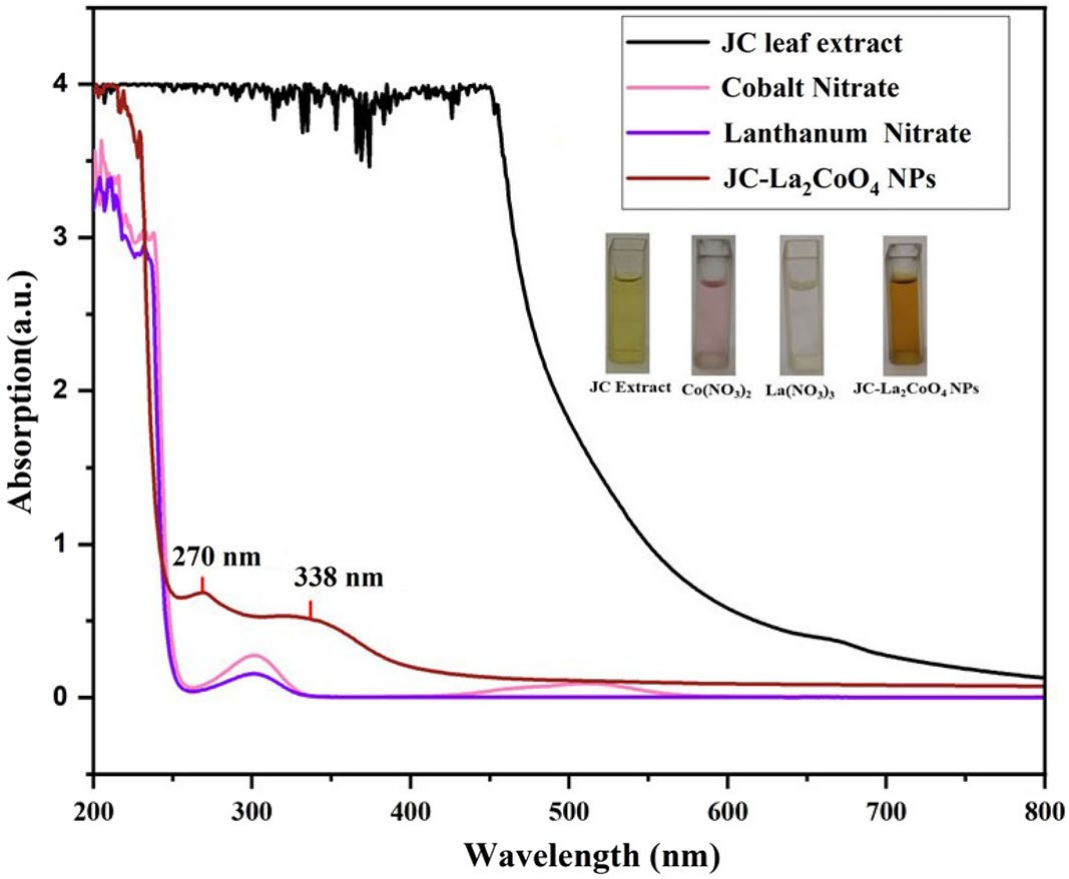
UV–visible spectra of JC leaf extract (black), Cobalt nitrate (pink), Lanthanum nitrate (purple) and JC-La_2_CoO_4_ NPs (brown).

The greenish yellow solution of JC-La_2_CoO_4_ NPs was obtained from a plant extract, Co(NO_3_)_2_ and La(NO_3_)_3_ solution. The formation of JC-La_2_CoO_4_ NPs was confirmed by UV–visible spectrophotometer. From the UV–visible spectroscopy, JC-La_2_CoO_4_ NPs have an absorption peak appears at 270 and 338 nm whereas no peaks observed in the mentioned bands for Co(NO_3_)_2_, La(NO_3_)_3,_ and Jatropha curcas extract solution shown in Fig. 2. Co(NO_3_)_2_ and La(NO_3_)_3_ solution absorption band was found at 300 nm.

### XRD analysis

**Figure 3:**
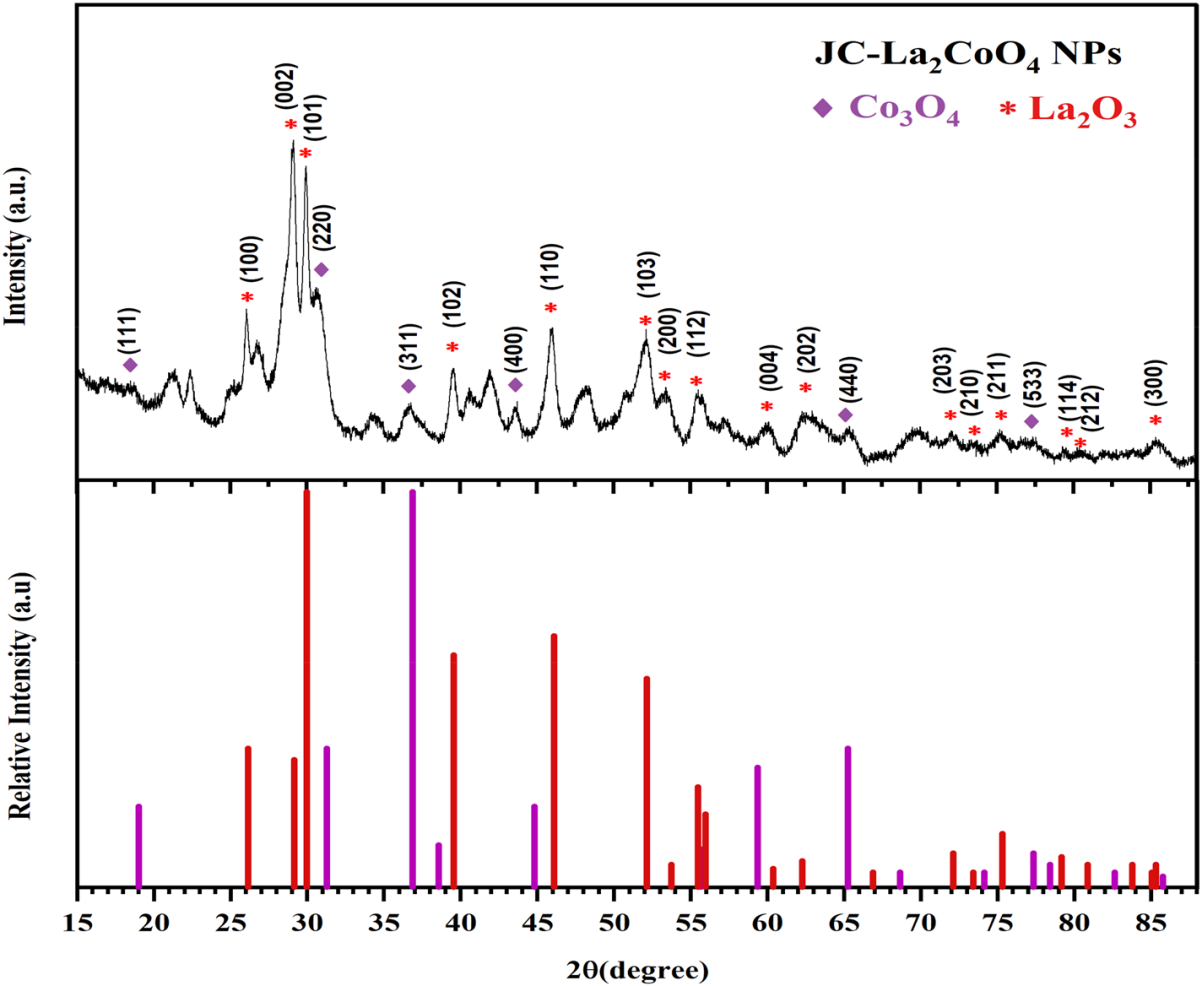
XRD Pattern of the JC-La_2_CoO_4_ NPs.

X-ray diffraction profile of JC-La_2_CoO_4_ NPs were obtained after calcination at 200 °C by using Panalytical Xpert^3^ powder with scanning angle (2θ) ranging from 15 to 90 degree (°) at 45 kV, 40 mA, by using Cu Kα radiation (λ = 1.5405 Å). XRD pattern illustrated in Fig. 3 confirm the diffraction pattern of the sample were taken and indexed by using the Joint Committee on Powder Diffraction Standards for cobalt oxide JCPDS card no. 00-042-1467, and shows comparative intense peak corresponding to the diffraction peaks at 2θ = 19.0, 31.2, 36.8, 43.7, 65.2, and 77.3° supported the prepared nanomaterials and exhibited the (hkl) values of (111), (220), (311), (400), (440), and (533) corresponding to the cubic structure of Co_3_O_4_^9,13^. Lanthanum oxide JCPDS card no.00-005-0602 corresponding to the diffraction peak at 2θ = 26.1, 29.1, 29.9, 39.5, 46.0, 52.1, 53.4, 55.4, 60.1, 62.4, 72.0, 85.6˚ and exhibited the (hkl) values of (100), (002), (101), (102), (110), (103), (200), (112), (004), (202), (203), (210), (211), (114), (212), (300) corresponding to the hexagonal structure of La_2_O_3_^10,47^, which is matched with good agreement of JC-La_2_CoO_4_. The highest intense peak of the composition indicates concentration of lanthanum is maximum compared to cobalt and supported the formation of La_2_CoO_4_. XRD pattern proves that JC-La_2_CoO_4_ is a spinel with perovskite structure. The average crystallite size of JC-La_2_CoO_4_ NPs is 11.3 nm estimated by using the Scherrer equation.

### FTIR analysis

**Figure 4:**
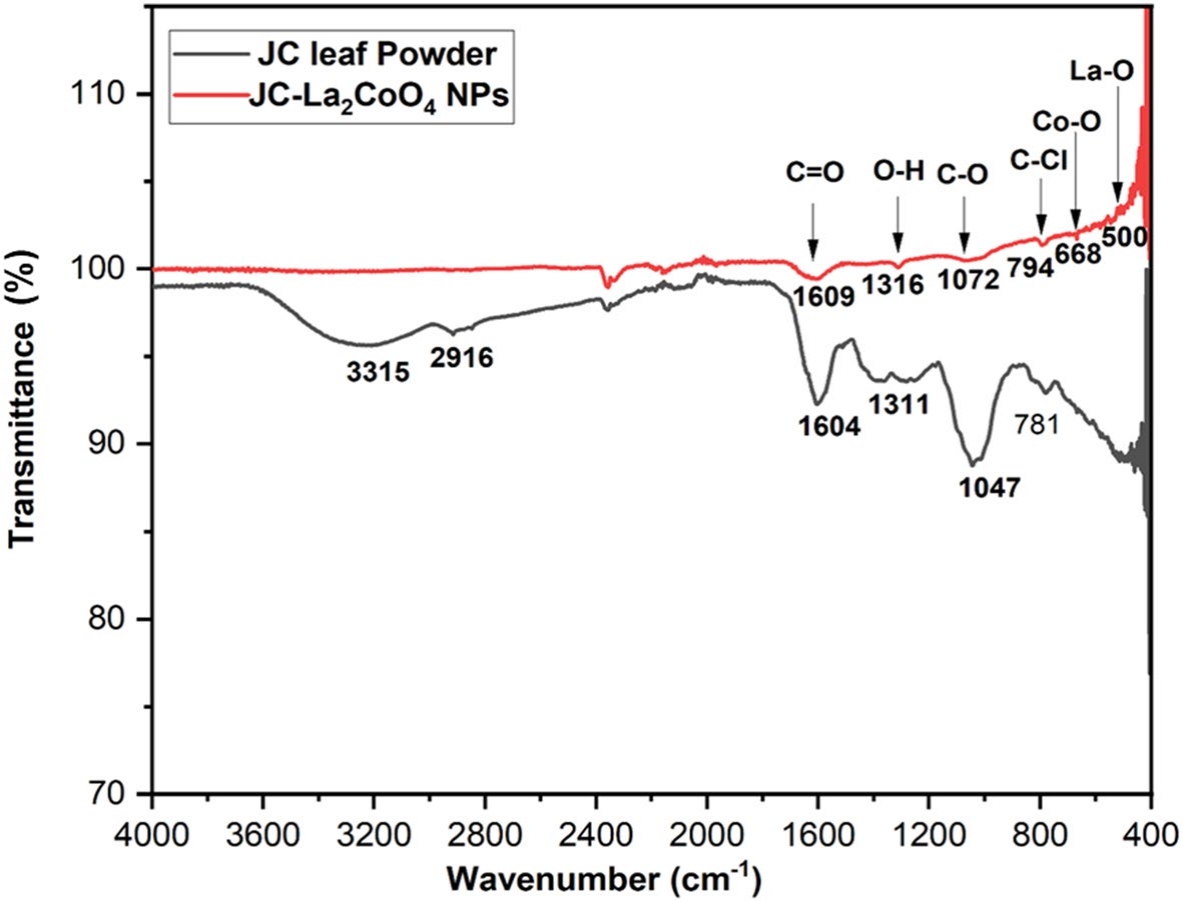
FTIR-Spectra of the JC-La_2_CoO_4_ NPs.

The vibrational property of the *Jatropha Curcas* leaf powder and JC- La_2_CoO_4_ are presented in Fig. 4. In the FTIR spectra shows significant peaks and wavenumbers and an interpretation of the possible functional groups. It also proofs the phytochemicals or functional groups in the JC leaf and are responsible for reducing and stabilizing the JC-La_2_CoO_4_ NPs. The characteristic stretching band appear at 500 cm^-1^ indicated the formation of La–O nanoparticles^48,49^. The band assigned at 668 cm^-1^ to the bridging vibration of O–Co–O bands^6,18^. The bands observed at 3315, 2916, 1604 and 1047, 1311 and 781 cm^-1^, respectively, for the presence of aqueous O–H, C-H, C-O, alcoholic O–H and C–Cl functional group of JC leaf powder. Simultaneously bands obtain in the JC-Co_2_LaO_4_ NPs at 1609, 1316, 1072, and 794 cm^-1^, corresponds to the C=O, O–H, C–O and C–Cl with good agreement and it might be responsible for the bio reduction of Co and La to the JC-Co_2_LaO_4_ NPs. The comparison study of the IR band observed between JC plant extract powder and JC-Co_2_LaO_4_ NPs shown in Table 2.

**Table 2:** The comparison study of the IR band observed in JC plant extract and JC-La_2_CoO_4_ NPs.

JC-leaf wavenumber (cm^-1^)	Probable functional group	JC-La_2_CoO_4_ NPs (cm^-1^)	Probable functional group
3315	Broad for O–H		
2916	Medium for C–H		
1604	C=O Stretch	1609	C=O Stretch
1311	O–H Bending	1316	O–H Bending
1047	Medium for C-O	1072	Medium for C–O
781	C–Cl Strong Bending	794	C–Cl Strong Bending
		668	Co–O
		500	La–O

### SEM and EDAX analysis of the JC- La_2_CoO_4_ NPs

**Figure 5:**
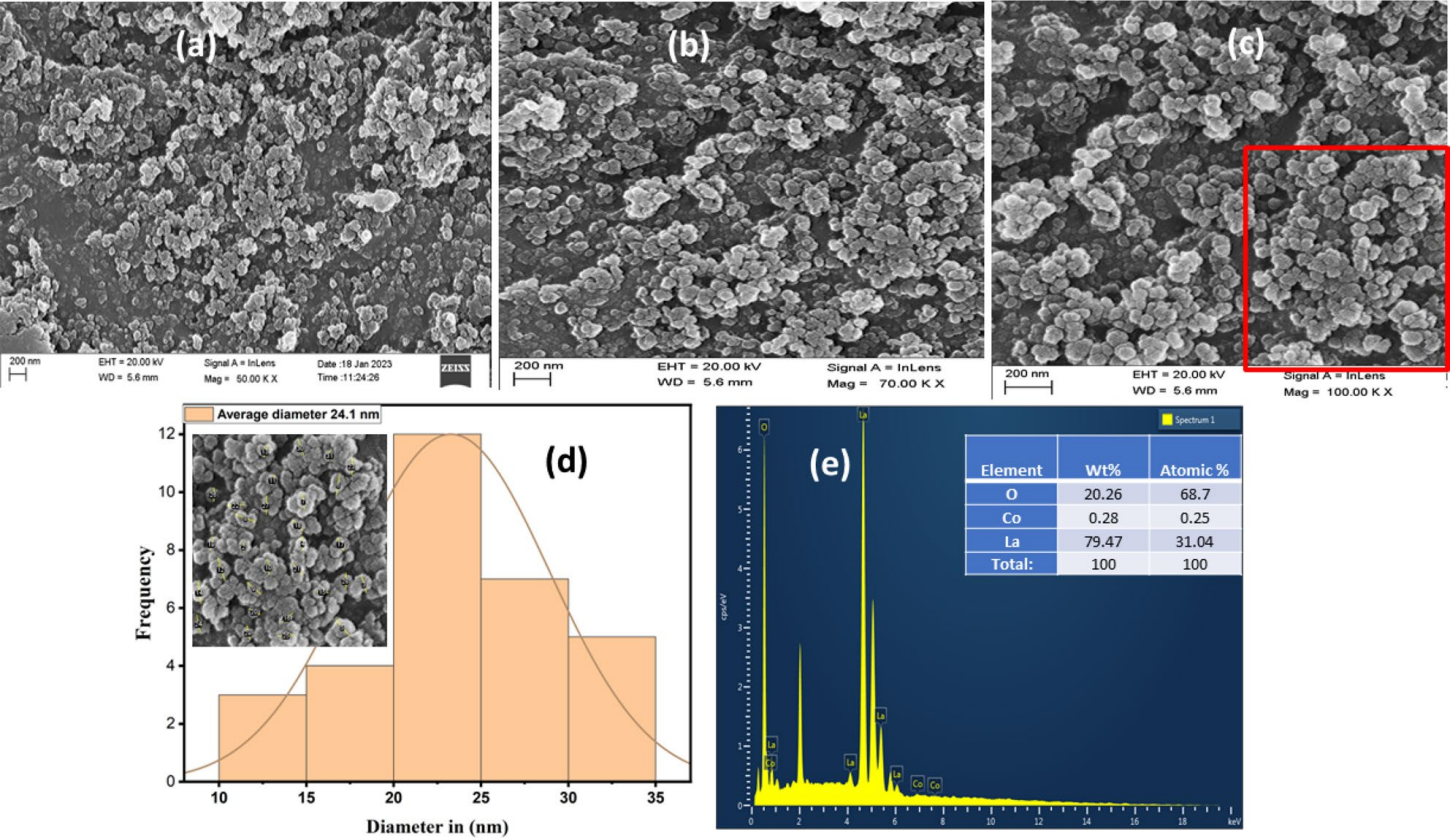
SEM image of the JC-La_2_CoO_4_ NPs at different magnification (**a**–**c**) and particle size distribution of C image in red box (**d**) and EDAX spectra (**e**).

The surface morphology of the prepared nanoparticles was examined using SEM analysis. Energy dispersive X-spectroscopy (EDAX) to identify the existing elements in the composite. Figure 5a–c represents the SEM image of the JC-La_2_CoO_4_ NPs at different magnification (50, 70 and 100KX), which indicates that the nanoparticles were well uniform and spherical shape and (d) showing the particle size distribution of c image in red colour. The constituents of the green synthesized JC-La_2_CoO_4_ NPs consist the elemental peaks for La at 4.5 keV, Co at 1 keV and O at 0.5 keV shows in Fig. 5e determined the atomic % of metals. The average grain sizes of the JC- La_2_CoO_4_ NPs is 24.1 nm estimated using ImageJ software and presented the histogram in Fig. 5d. The sample agglomerates of NPs with spherical shape have very fine particle prepared by green method using leaf extract of *Jatropha curcas*.

### XPS analysis of the JC-La_2_CoO_4_ NPs

**Figure 6:**
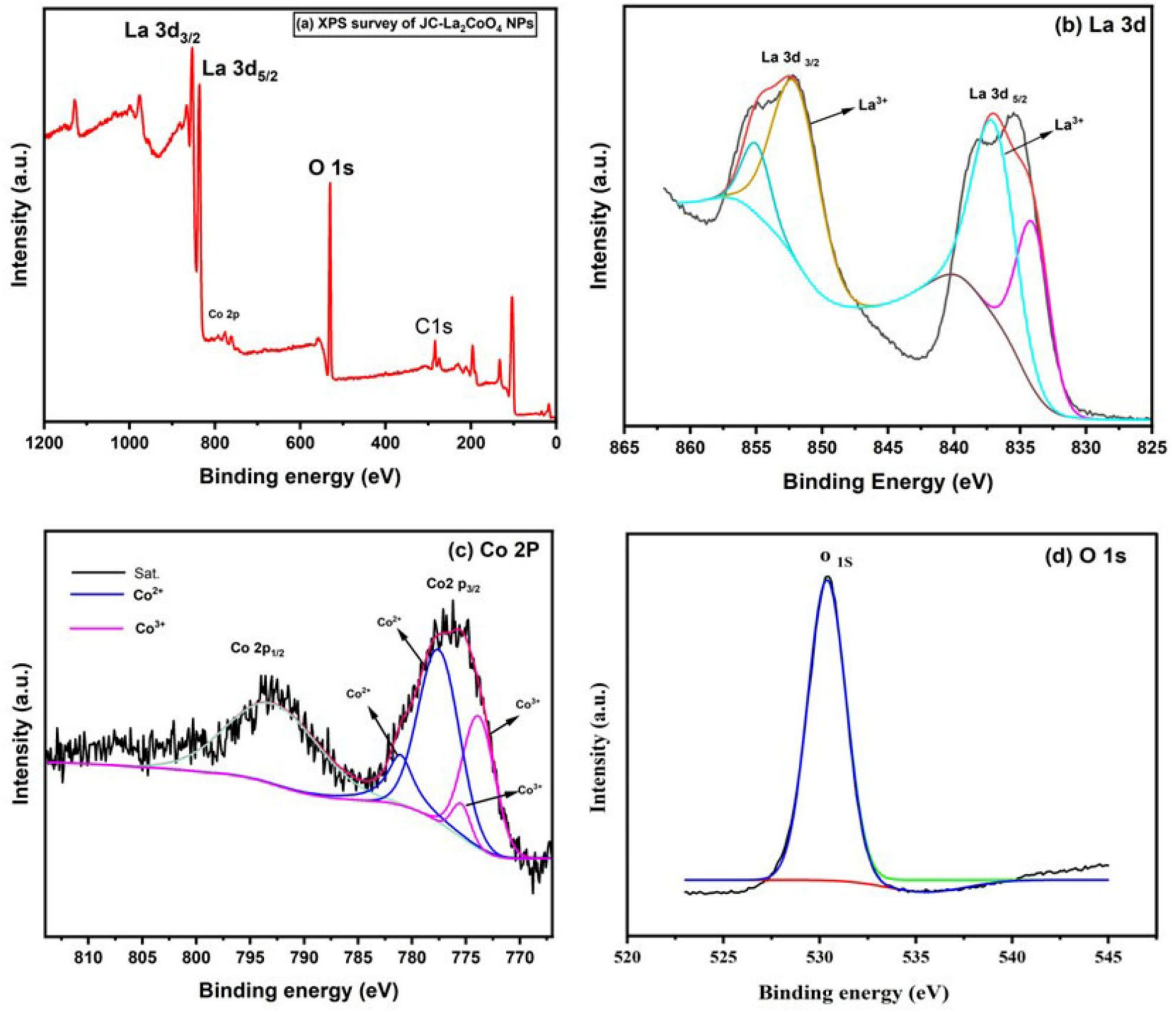
XPS Spectra for the (**a**) XPS-Survey analysis of the JC-La_2_CoO_4_ NPs, (**b**) La 3d, (**c**) Co 2P and (**d**) O 1 s of the JC-La_2_CoO_4_ NPs.

Surface oxidation state and chemistry of La and Co ions in JC-La_2_CoO_4_ were further investigated by using the core-level and satellite X-ray photoelectron spectroscopy (XPS). The binding energy (eV) and features of Co2p, La3d and O1s spectra shown in Fig. 6. Figure 6a display the hole spectra of JC-La_2_CoO_4_ and assign the signals of La3d, Co2p, O1s and C1s. The lanthanum elements represented by two major peak corresponds to La3d state existence in binding energy at 852.3 eV for La3d_3/2_ and 835.5 eV for La3d_5/2_ respectively shown in Fig. 6b and the splitting of La is good agreement with La3d spectrum of La-based perovskites and existence of + 3 oxidation state of La^48–50^. Figure 6c represent the binding energy spectra of Co elements having two peaks Co2p state existence in 793.6 eV for Co2p_1/2_ and 776.3 eV for Co2p_3/2_, respectively. The lower binding energy with intense XPS signals of the Co2p_3/2_ indicates of Co^3+^ ions, whereas higher binding energy with the low-intensity signals of Co2p_3/2_ can be assigned to the Co^2+^ ions^51,52^. At the XPS spectra of JC-La_2_CoO_4_, the core-level signals of Co^2+^ ions show in higher binding energy site more intense XPS signals relative to those of Co^3+^ ions, and a high-intensity peak of metallic Co appears at ~ 777.8 eV suggest + 2 oxidation state^53–55^. Which support the binding energy of O is located in 530.4 eV and valency is O1s shown in Fig. 6d. Therefore, the XPS spectra supported and conclude that the probable composition is JC-La_2_CoO_4,_ which satisfied the valency and total charges are balanced in the composition.

### Optical properties

**Figure 7:**
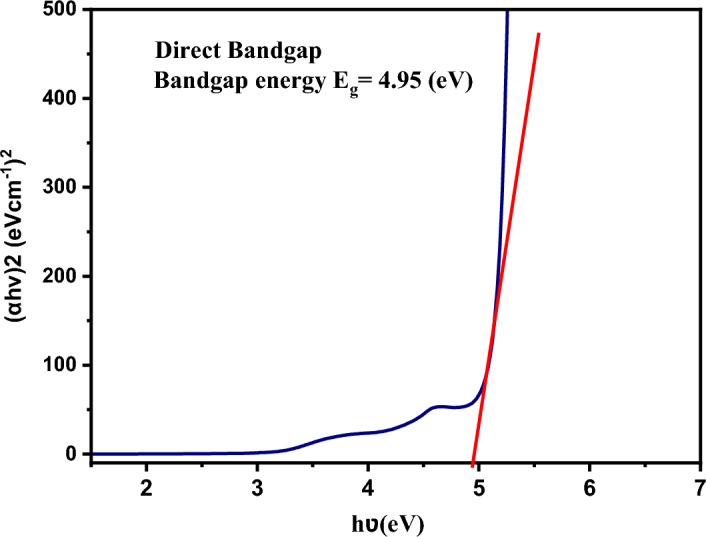
Band gap energy of JC-La_2_CoO_4_ NPs.

The UV–visible spectrum of the JC-La_2_CoO_4_ is displayed in Fig. 7. The interaction of the JC-La_2_CoO_4_ the band edge appearing in UV–visible spectrum at 270 and 338 nm. The optical absorption study of the JC-La_2_CoO_4_ NPs revealing that electronic transition, band gap energy and luminescent property^11,56^. Band gap energy was calculated by using Tauc’s relation (Eq. 1).

1 $$(\alpha hv)^{n} = A(hv - E_{g} )$$

where α: represents the absorption coefficient, A: is a constant, E_g_: is showing optical band gap, n: is exponent that depends on transition, h: is symbol of plank\rsquo s constant

**Figure 8:**
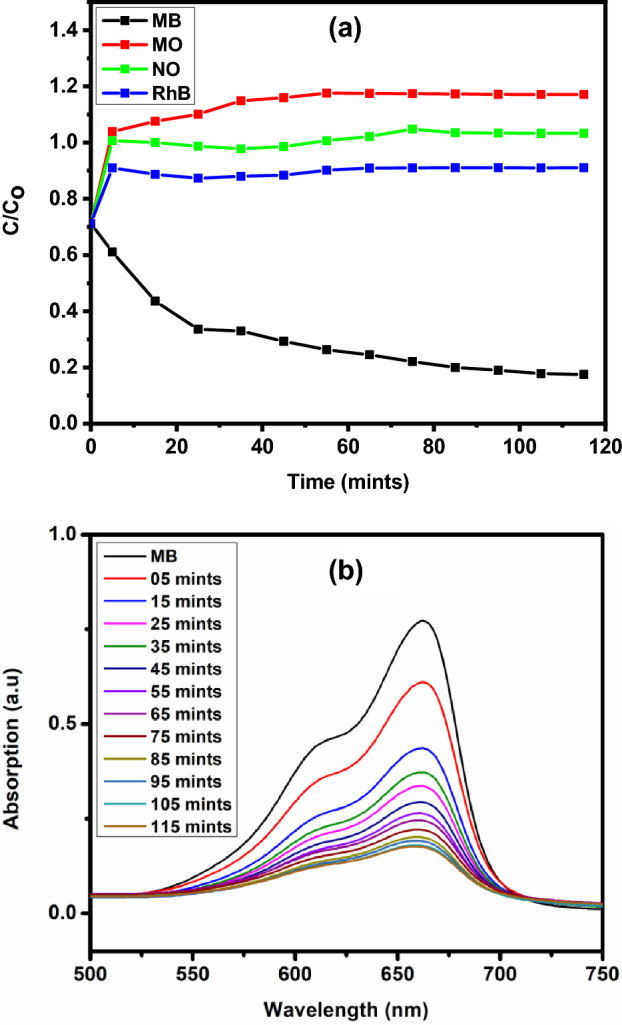
(**a**) Degradation of different dyes in presence of JC-La_2_CoO_4_ and sunlight, and (**b**) UV–visible Spectra of MB in presence of JC-La_2_CoO_4_ NPs.

The optical energy band gap energy of JC-La_2_CoO_4_ is 4.95 eV calculated from Fig. 8, which appears through extrapolating the linear portion of the curve to (αhʋ)^2^ = 0 and indicates its semiconductor properties and support the study of catalytic activity. Indirect band gap value is also calculated and showing in Figure S1.

### Photocatalytic activity

**Figure 9:**
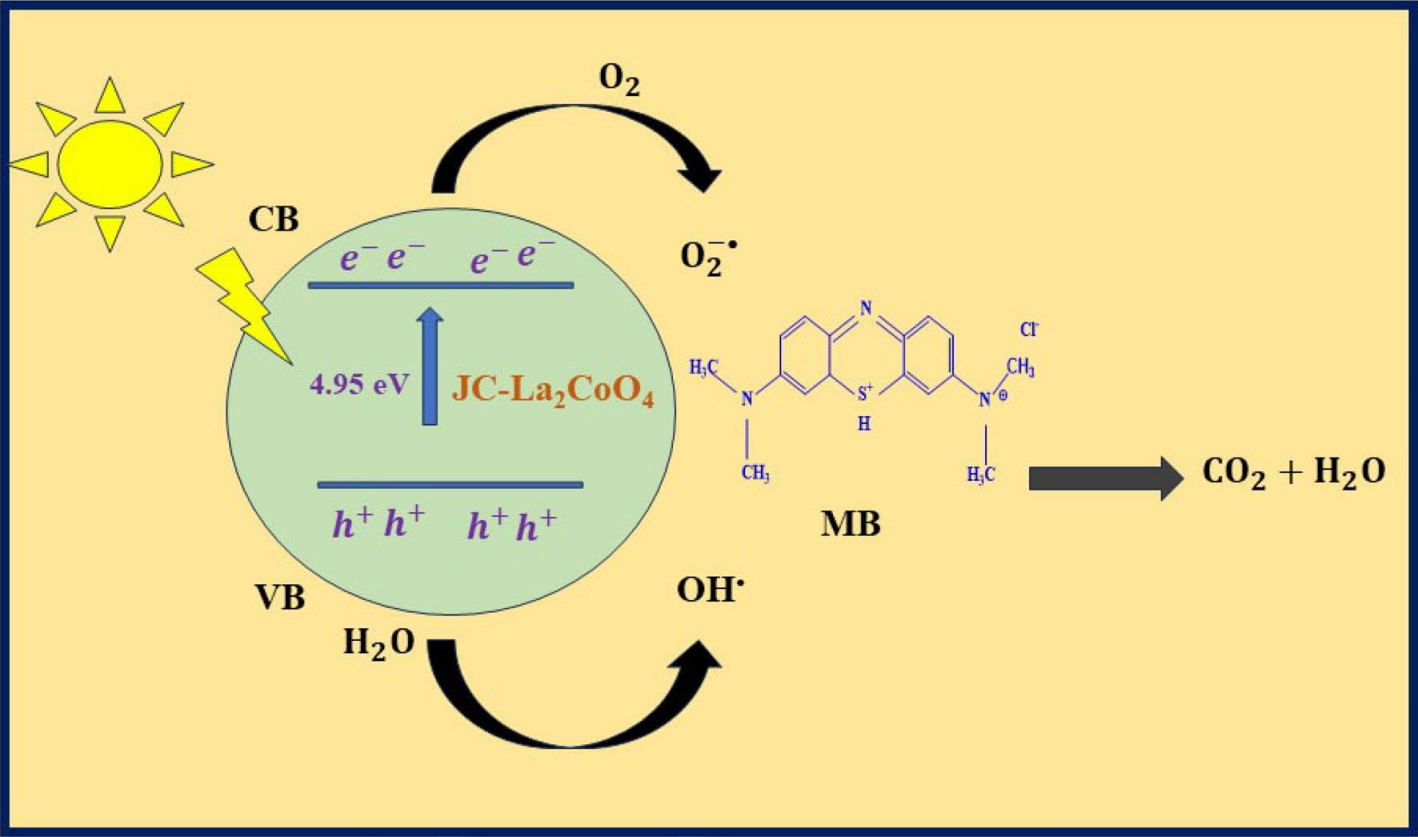
Probable mechanism for degradation of MB in presence of JC-La_2_CoO_4_.

Photocatalytic experiments were conducted using JC-La_2_CoO_4_ NPs in presence of sunlight aqueous solution of different dyes like NO, MB, MO and RhB. The reactions were performed by adding JC-La_2_CoO_4_ (0.1 g) into each set of a 20 mL solution of MB (3 mg/L) dyes. Before the degradation process solution agitated in the dark for 15 mint to established adsorption/desorption time is 20 mint to achieve equilibrium between MB solution and nanoparticle. The most prominent result was found in case of MB, which is faster degraded compared to others dyes with small time is shown in Fig. 8a. The degradation of MB in presence of JC-La_2_CoO_4_ NPs was examined by UV–VIS spectrophotometer (UV-1800, Shimadzu) after 10 min interval shown in Fig. 8b. The initial absorbance of MB is about 0.712 at 662 nm and it takes 115 min for complete degradation after that the degradation of MB is almost constant. The rate constant of JC-La_2_CoO_4_ is 56.73 × 10^–3^ after 50% degradation of MB with respect to irradiation time. Therefore, JC-La_2_CoO_4_ NPs shows good catalytic activity against MB compared to other dyes. The probable mechanism for degradation of MB is shown in Fig. 9. It interprets that in presence of sunlight JC-La_2_CoO_4_ was activated by absorbing specific wavelength of sun light and creates electron/hole pair in the valance band. This electron is move from valance band to conduction band and generate hole pair in the valance band. Simultaneously electron populated in the conduction band because of band gap of JC-La_2_CoO_4_ is 4.95 eV and electron easily move from valance band to conduction band. The oxidation and reduction reaction will occur at valence and conduction band respectively. The dye molecule at first converted to dye radical cation by absorbing photon after that superoxide _2_ (O_2_^∙−^) and hydroxyl (∙OH) radical was formed from oxygen and water molecule and finally MB degraded into CO_2_ and water. UV–Vis. Spectra of MO, NO, and RhB shows in Figure S2 and dye degradation efficiency of MB, RhB NO and MO, summarize in the Table S1.

#### Effect of Catalytic dosage and initial concentration of dye on the dye degradation

**Figure 10:**
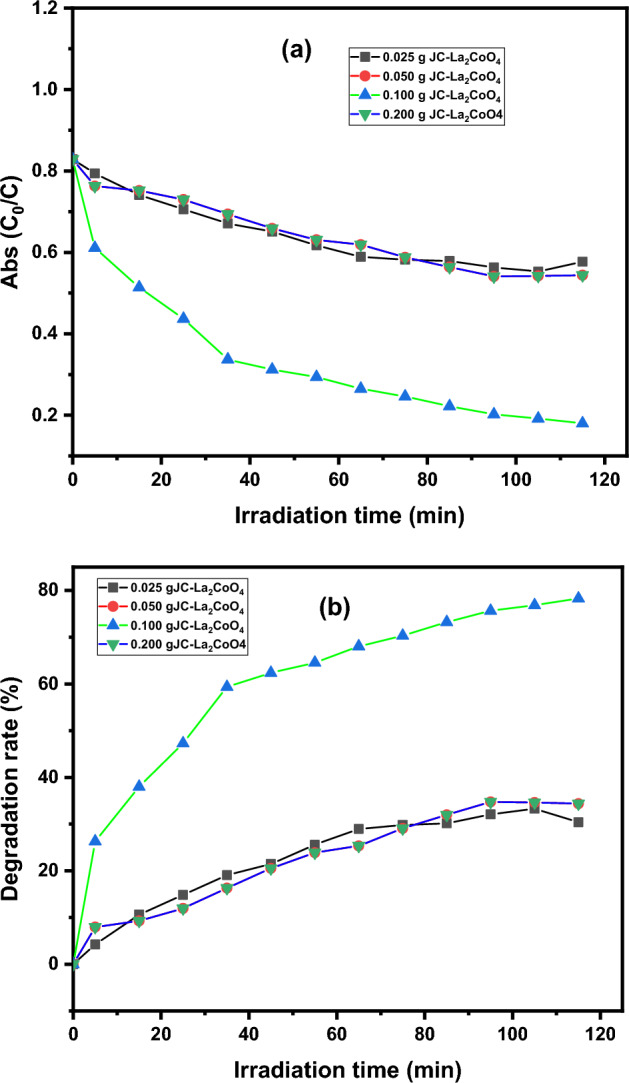
(**a**) Change in the concentration of MB dye in different catalytic dosage in presence of JC-La_2_CoO_4_. (**b**) Changes in the degradation rate of MB dye in different catalytic dosage in presence of JC-La_2_CoO_4_.

For catalytic efficiency and to avoid the wasting of photocatalyst, it’s necessary to optimize the amount of catalyst in the photocatalytic degradation process. The effect of the catalyst dosage of JC-La_2_CoO_4_ was investigated in the degradation of MB using 0.025 g,0.05 g, 0.1 g and 0.2 g of catalyst for 115 min, which is shown in Fig. 10a. The degradation of MB dye increased as the quantity of catalyst increased from 0.025 to 0.2 g. which is shown in Fig. 10 b. As the amount of catalyst dose increased, the amount of adsorbed dye on the surface of the catalyst increased. Now the adsorbed dye molecule promptly reacts with ROS (reactive oxygen species)^57–59^. In the present study increased the catalyst dosage from 0.025, 0.05, 0.1, and 0.2 g/20 mL, the degradation percentage of MB dye initially increased which may be attributed to the active ROS sites generation. But in higher dosage of catalyst 0.2 g degradation percentage of MB dye decrease, and it could be due to the decrease in photon penetration on the catalyst surface block in solution which leads to a decrease the formation of ROS^60^.

**Figure 11:**
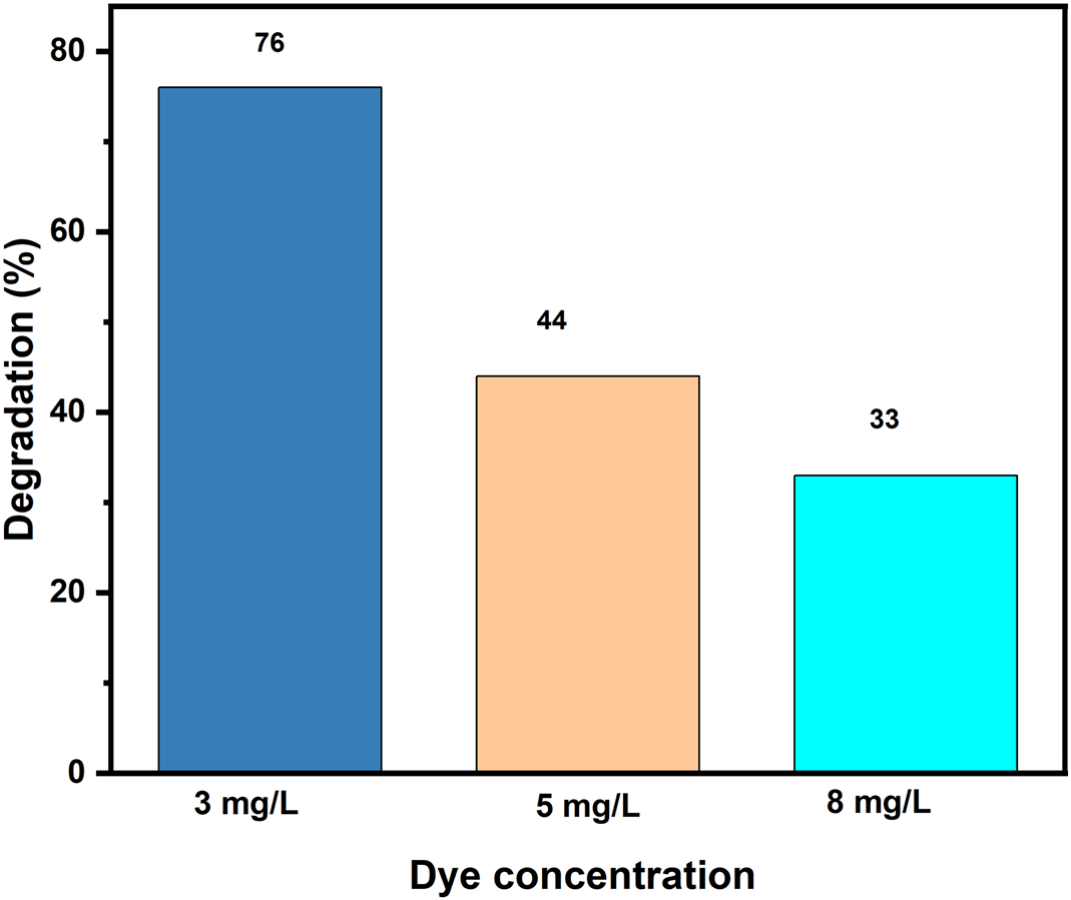
Effect of initial dye concentration on the photodegradation of JC-La_2_CoO_4_ NPs.

The effect of the initial dye concentration of MB on the dye degradation efficiency was studying in presence of catalysts. The concentration of dye varying from 3 to 8 mg L^-1^ with 0.1 g photocatalyst JC-La_2_CoO_4_ and percent degradation is shown in Fig. 11.

Figure 11 shows the effect of the dye concentration on the performance of the photocatalyst. The rate of photocatalytic degradation decreases from 76 to 33%. This degradation percentage occurred with an increase in dye concentration because dye concentration blocks the surface-active sites of photocatalyst, inhibiting the process for ROS generation and turn decreasing the degradation efficiency^61,62^. The maximum degradation efficiency was found in the 3 mg/L^-1^ solution of MB, and therefore MB dye was chosen as the optimum concentration for further degradation process.

#### Effect of pH on dye degradation efficiency

**Figure 12:**
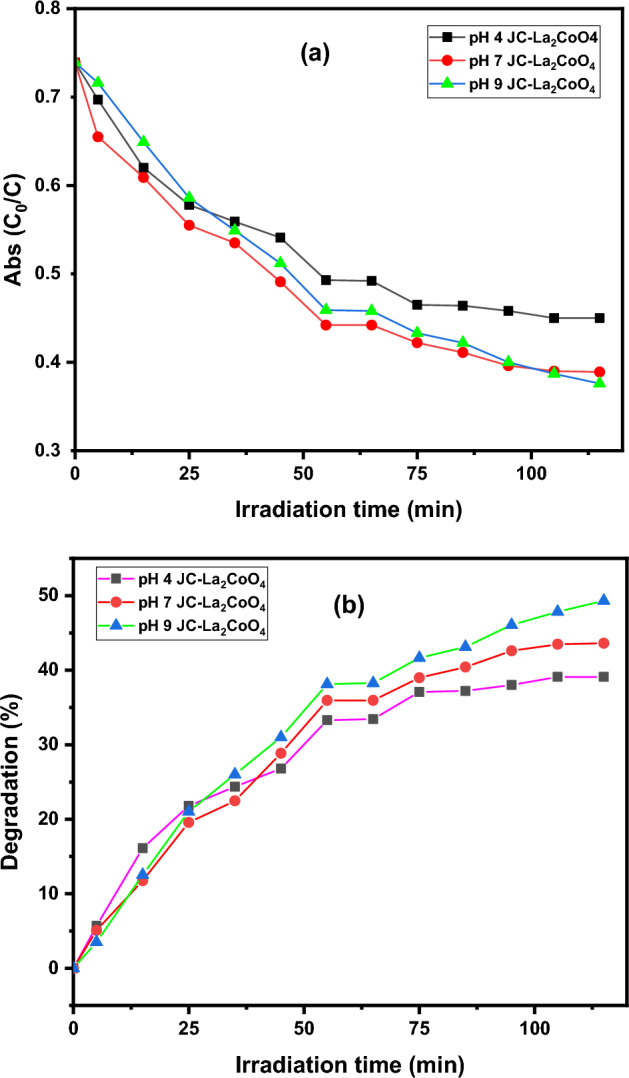
(**a**) Change in the concentration of MB dye at different pH value in presence of JC-La_2_CoO_4_. (**b**) Changes in the degradation rate of MB dye at different pH value in presence of JC-La_2_CoO_4._

The effect of the pH on the photocatalytic degradation of MB dye was investigated in the presence of JC-La_2_CoO_4_ photocatalyst in sunlight which is shown in Fig. 12a. The pH of the reaction from pH = 4, 7 and 9. Adjustment of pH with in the above range by using 0.1 M solution of HCl and NaOH. JC-La_2_CoO_4_ photocatalyst giving better catalytic activity at pH 9 which is shown in Fig. 12b. As the resulting value nearly same at pH 4,7,9 is more favorable for the degradation of MB dye^54,63^.

#### The Effect of active species scavenger test

**Figure 13:**
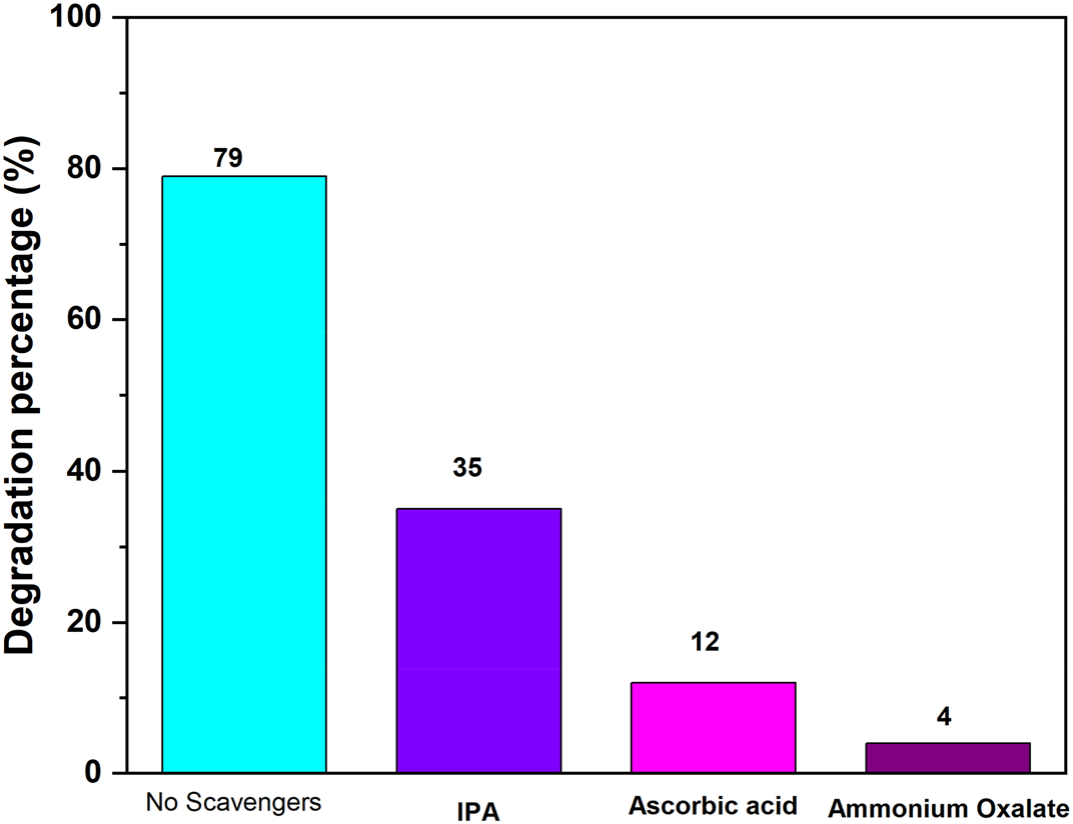
The role of active species on the MB photocatalytic dye degradation.

To evaluate and understand the active species, i.e., (O_2_^∙−^)(superoxide), h^+^ (holes), and ˙OH (hydroxyl ion) were used to study the photocatalysis mechanism which is shown in Fig. 13. For this purpose, isopropyl alcohol (IPA,1 mM) was used as ˙OH, ascorbic acid (AA, 1 mM) used for trapping _2_(O_2_^∙−^) and ammonium oxalate (AO, 1 mM) used as a h^+^ scavenger. In the absence of any active species degradation rate of MB was 79%. With the addition of IPA, AA, AO degradation of MB was decrease about 35%,12%,4% respectively. The addition of all the species contribute to the degradation of MB dye.

The rate of degradation in presence of IPA and AA rate is highest, therefore the ˙OH, and O_2_^-^ species play a key role for the degradation^60,64,65^.

#### Recyclability and stability of the JC-La_2_CoO_4_

**Figure 14:**
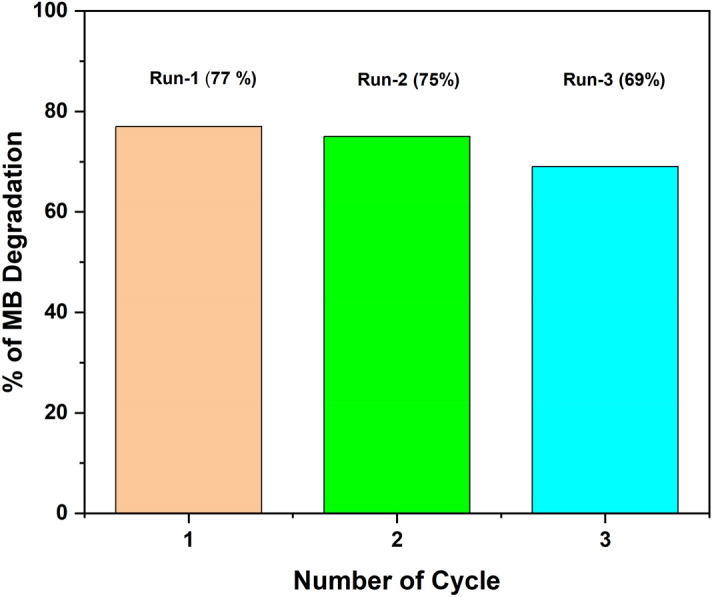
Photocatalytic degradation reusability performance of JC-La_2_CoO_4_.

Recyclability and stability is an important factor after the degradation process, for this prediction photocatalyst was investigated for reusability by subjecting it to three consecutive experiment cycle under the same condition up to 115 min. Figure 14 attribute to the effect of reusability test to MB degradation, the first two test efficiency rate of degradation nearly the same (77% to 75%). During the third test degradation rate 69%. The Reduction in degradation efficiency it might be due to the loss of photocatalyst in each cycle and active site blockage^19,66^.

The stability of the reused photocatalyst after repeated cycle was characterized by XRD pattern of JC-La_2_CoO_4_, which is shown in Figure S4. After the third cycle peaks showing that there is no structural change.

### Magnetic (SMMs) study of the JC-La_2_CoO_4_ NPs

#### DC magnetic study

**Figure 15:**
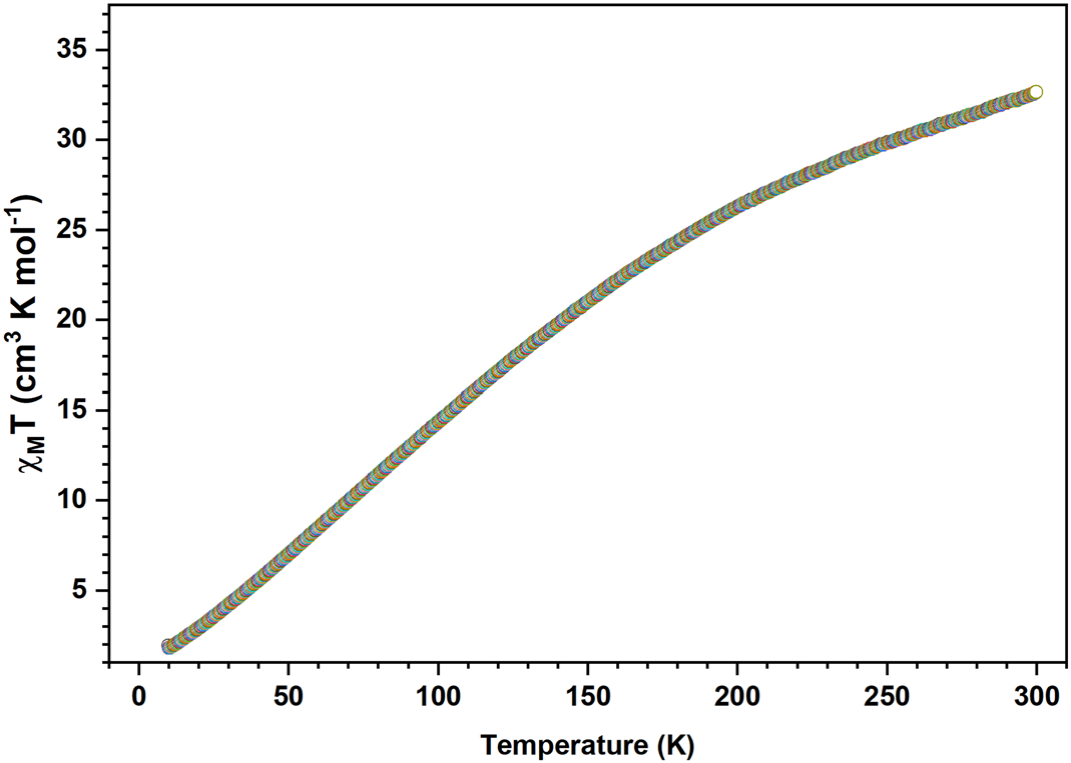
DC magnetic susceptibility χ_M_T vs T plot of the JC-La_2_CoO_4_ NPs.

The DC magnetic susceptibility of the JC-La_2_CoO_4_ NPs the temperature dependences **χ**_**M**_ and **χ**_**M**_**T** are depicted in Fig. 15. Magnetic susceptibility as investigated under 10–300 K temperature and applied field is a 1000 Oe (0.1 T). The value of **χ**_**M**_**T** 32.66 cm^-3^ mol^-1^ K, this value is the contribution of Co (II) ion ^4^F_9_/_2_ (S = 3/2, L = 0, g = 2) and La (III) ^1^S_0_ (S = 0, L = 0, g = 1)(also support from XPS). Upon cooling, the value of **χ**_**M**_**T** decreases monotonically to attain a minimum value 1.55 cm^3^ mol^-1^ K at 10.3 K, which is indicative of the existence of antiferromagnetic coupling.

**Figure 16:**
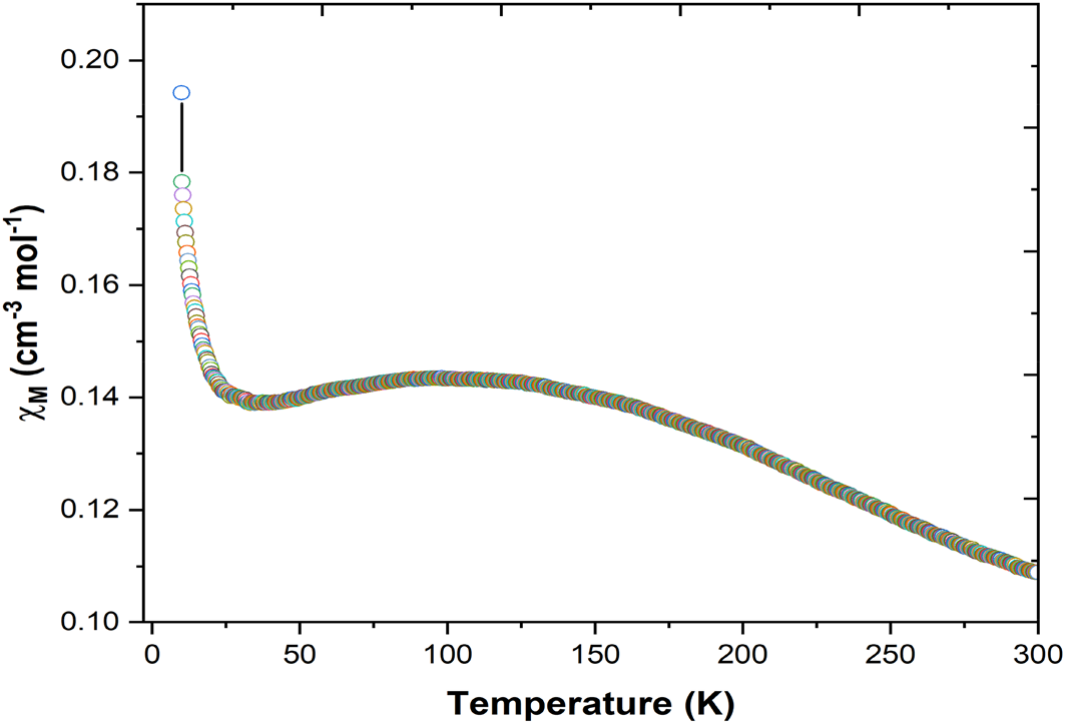
χ_M_ vs T plot for the JC-La_2_CoO_4_ NPs.

**Figure 17:**
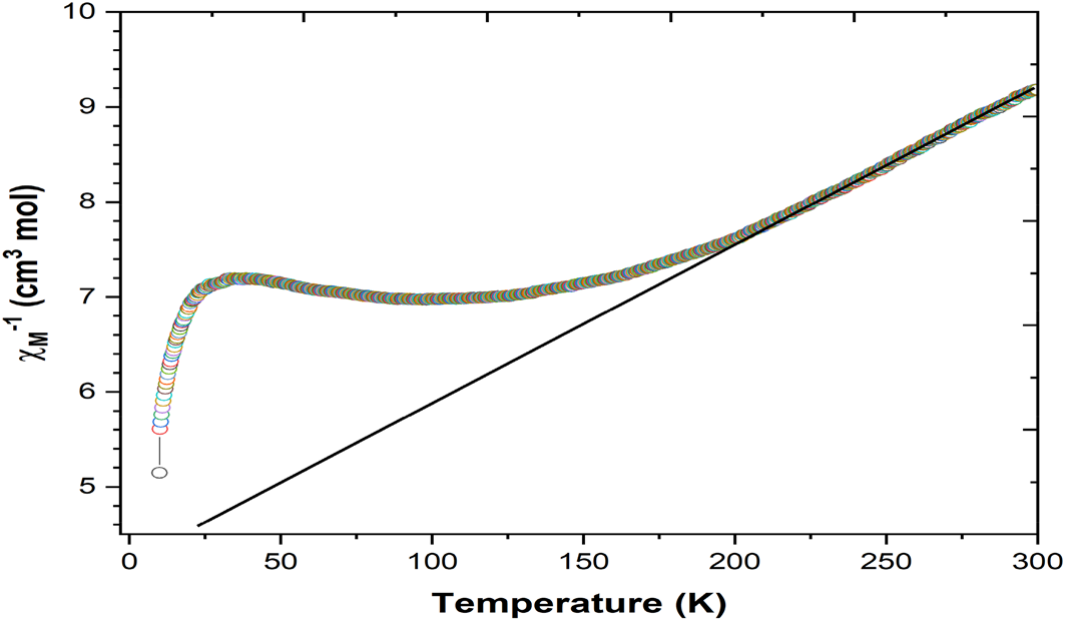
Plot of χ_M_^-1^ vs T plot for the JC-La_2_CoO_4_ NPs.

In the Fig. 16 as temperature decrease from 300 K, the **χ**_**M**_ value increase, reaching a maximum 0.143 cm^3^ mol^-1^ at 94 K and then decreases slightly reaching a value 0.138 cm^3^ mol^-1^ at 38 K. Upon further cooling, the **χ**_**M**_ value increase again to 0.178 cm^3^ mol^-1^ at 10 K. Figure 17 showing the temperature dependence of **1/χ**_**M**_ at temperature above 195 K has been fitted by the Curie–Weiss law^4,15^.

#### AC magnetic study

**Figure 18:**
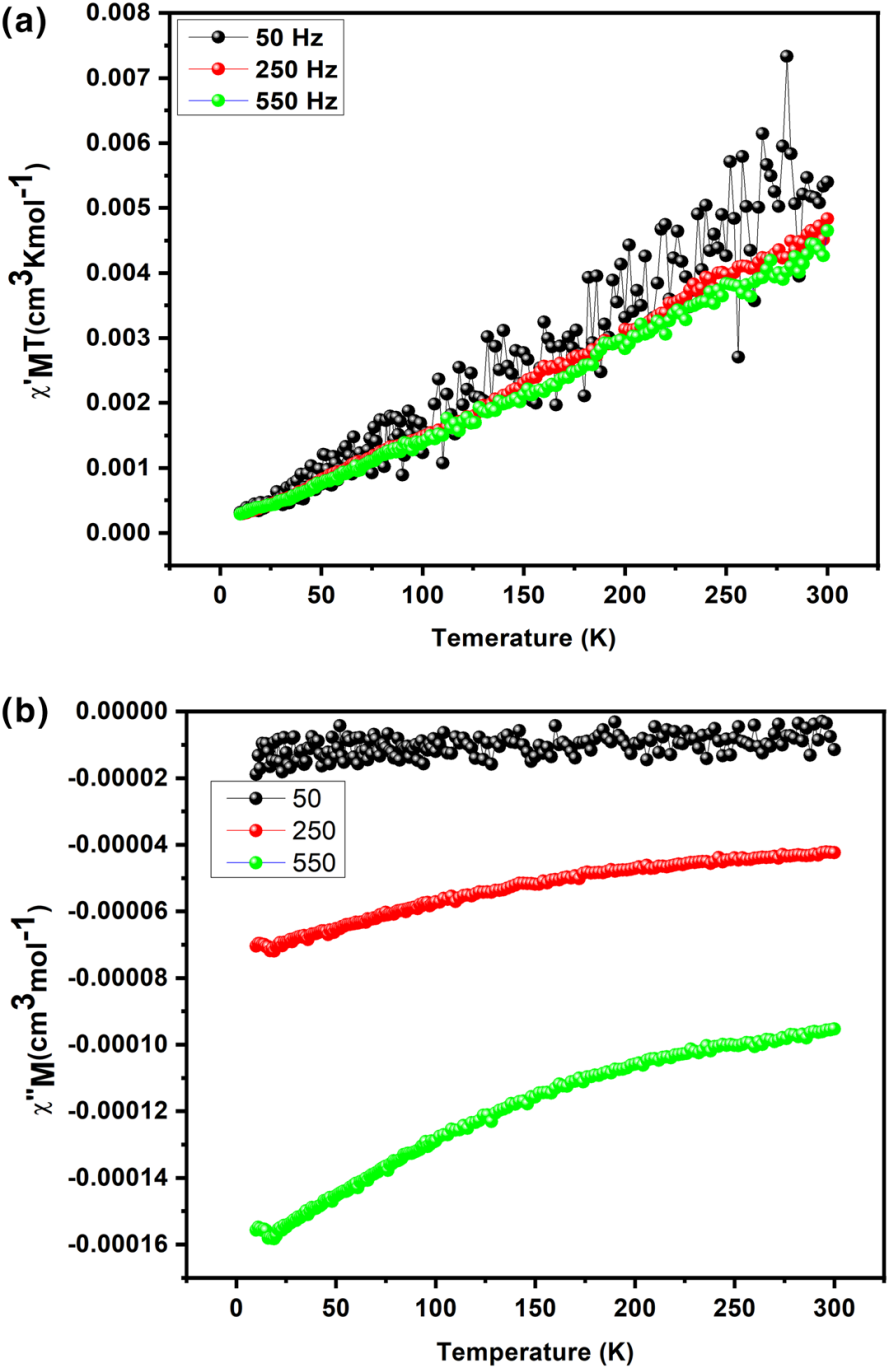
(**a**) AC Susceptibility in-phase χ' M in phase plot for the JC-La_2_CoO_4_ NPs. (**b**) AC Susceptibility out-phase χ''M out-of-phase plot for the JC-La_2_CoO_4_ NPs.

AC susceptibility (in phase and out phase) studies have been conducted for the JC-La_2_CoO_4_ NPs in between 1.8 and 15 K in a zero applied field with 3.5Oe driving field to investigate for slow magnetic relaxation, i.e., SMM behaviour. The AC susceptibility studies for nanoparticles have been performed at various frequencies such as 50, 250 and 550 Hz and a plot of χ_M_T versus temperature (in phase and out phase) is presented in Fig. 18a and b.

The ac-in-phase susceptibility of naoparticles are in good agreement with the dc data at the same temperature. χ_M_^'^T value is significantly increased with increasing the temperature, having a maximum value of 0.0054 cm^3^ Kmol^-1^ at 300 K (Fig. 18a). The frequency dependent rise in the out-of-phase susceptibility is observed as a peak tail, indicating nanoparticles displays behaviour characteristic of a SMM (Fig. 18b and Table S2)^67^.

### Novelty of the work

In the past, researchers have synthesized La_2_CoO_4_ using various methods, including Spray Flame and Sol–gel techniques^68,69^. However, our research marks the first instance of synthesizing La_2_CoO_4_ by using environmentally friendly, green methods. While other research groups have focused on producing nanoparticles of cobalt (Co) and lanthanum (La), by sol–gel or other approach. Therefore, our approach stands out due to its uniqueness. What's more, until now, no nanoparticles have exhibited the dual properties of acting as both photocatalysts and single-molecule magnets (SMMs). The literature reviews presented in the Table 3 underscore the innovative nature of our work.

**Table 3:** Comparative study.

Catalyst	Synthesis route	Application	References
La_2_CoO_4+δ_	Spray flame synthesis	Bisphenol degradation	^68^
La_2_CoO_4+δ_	Sol–gel method	Bisphenol degradation	^69^
La_1.2_Sr_0.8_CoO_4_	Solid state method	Ferromagnetic	^70^
LaCoO_3_	Sol–gel method	Methylene Blue and Ortho-Toluidine Blue degradation	^71^
LaCoO_3_	Proteic method	Methyl orange, Rhodamine B removal	^72^
CoO/Co_3_O_4_	Plant-extract-derived	Ferromagnetic, cytotoxicity	^9^
Co_3_O_4_	Sol–gel method	Ferromagnetic	^73^
Co_3_O_4_	Hydrothermal method	Antiferromagnetic	^74^
La_2_Ti_2_O_7_, (LTO)	Molten salt technique	Rhodamine B degradation	^75^
JC-La_2_CoO_4_	Plant-extract derived	Methylene Blue Degradation, Antiferromagnetic	Present study

## Conclusion

In the summary, first time report the bimetallic magnetic JC-La_2_CoO_4_ NPs was synthesized from aqueous leaves extract of *Jatropha curcas* through green approach and characterized by different spectroscopic technique. The JC-La_2_CoO_4_ NPs was stable up to six months due to presence of both capping and reducing agent in the leaves extract to stabilize the metal nanoparticles. The leaves extract contained (–COO^-^, –NH_2_ and –OH) groups where –OH and –NH_2_ groups involved to reduction of metal ion and –COO^-^ group strongly bind to the surface of NPs. JC-La_2_CoO_4_ NPs are semiconductor materials for which it degraded the methylene blue (MB) in presence of sunlight. Both spectroscopy studies (XPS and DC Magnetic) prove the La and Co is present in + 3 and + 2 oxidation state and support the formation of La_2_CoO_4_ spinel perovskite structure. JC-La_2_CoO_4_ NPs have antiferromagnetic interactions and the value of C is 0.842 cm^3^ K mol^-1^ by Currie-wises law. From DC and AC magnetic studies JC-La_2_CoO_4_ NPs shows good SMM properties. JC-La_2_CoO_4_ NPs may used as catalyst in organic transformation reaction. We will work on it in future.

### Supplementary Information

Supplementary Information 1.

Supplementary Information 2.

Supplementary Information 3.

Supplementary Information 4.

Supplementary Information 5.

Supplementary Information 6.

Supplementary Information 7.

## Data Availability

All data generated or analysed during this study are included in this submitted article and its supplementary information files.
