# Supplementary material for: Biosynthesis of JC-La2CoO4 magnetic nanoparticles explored in catalytic and SMMs properties
Source: Sci Rep. 2023 Dec 13;13:22122. doi: 10.1038/s41598-023-47852-9 (PMC10719267; doi:10.1038/s41598-023-47852-9)
Supplement: Supplementary file 7 — Supplementary Information 7. [file 41598_2023_47852_MOESM7_ESM.docx]

**Biosynthesis of JC-La_2_CoO_4_ Magnetic Nanoparticles Explored in Catalytic & SMMs properties**

**Nilesh Satpute^a^, Mithun Kumar Ghosh^a,b^, Aparna Kesharwani ^a^, Tanmay Kumar Ghorai^a,^***

***^a^Nanomaterials and Crystal Design Laboratory, Department of Chemistry, Indira Gandhi National Tribal University, Amarkantak, Madhya Pradesh 484887, India***

***^b^Department of Chemistry, Govt. College Hatta, Damoh, Madhya Pradesh 470775, India***

**Supporting Information**

Figure S1. Indirect band gap calculation of JC-La_2_CoO_4_ NPs.

**Figure S1. Indirect Band gap energy of JC-La_2_CoO_4_ NPs**


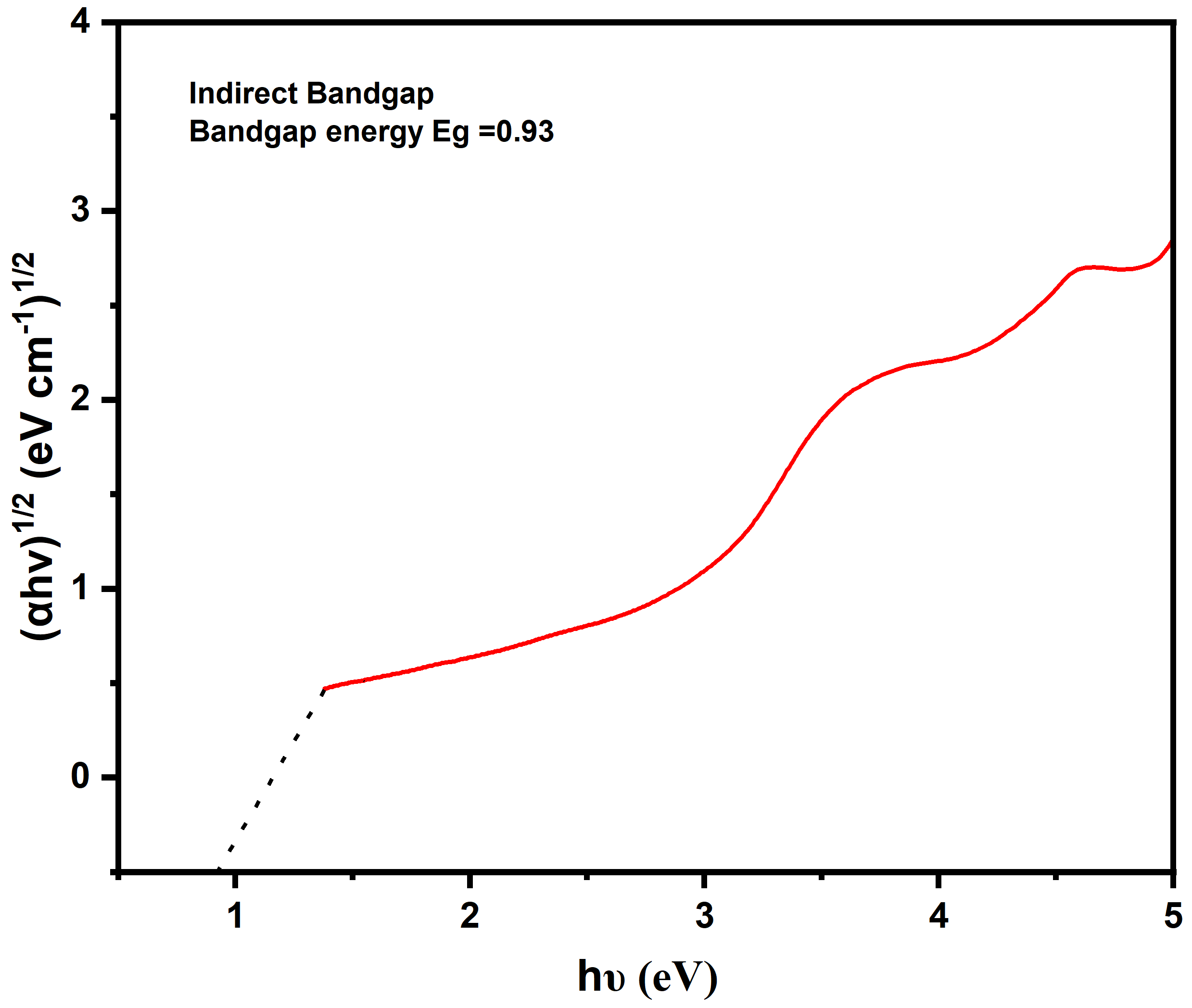

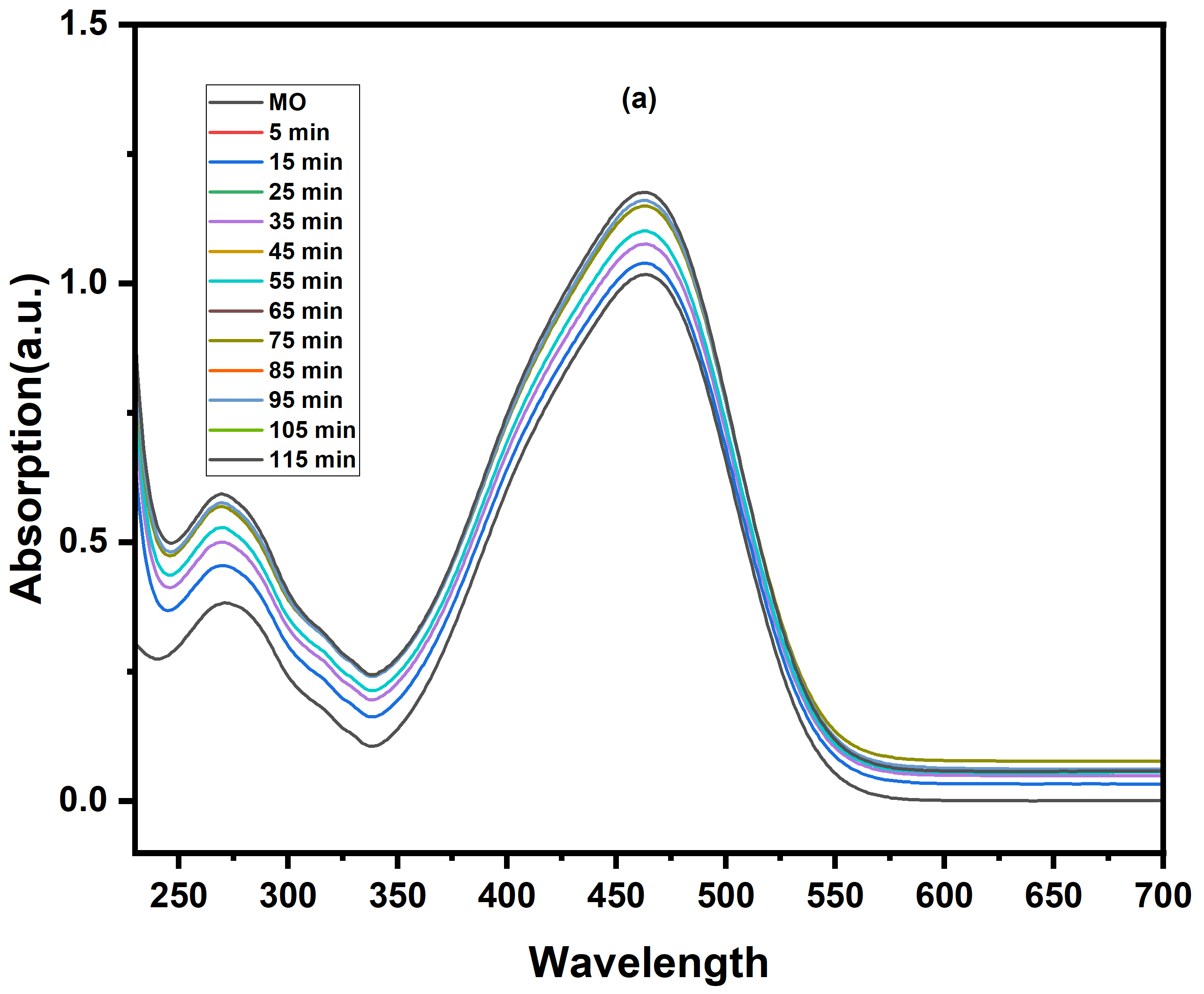

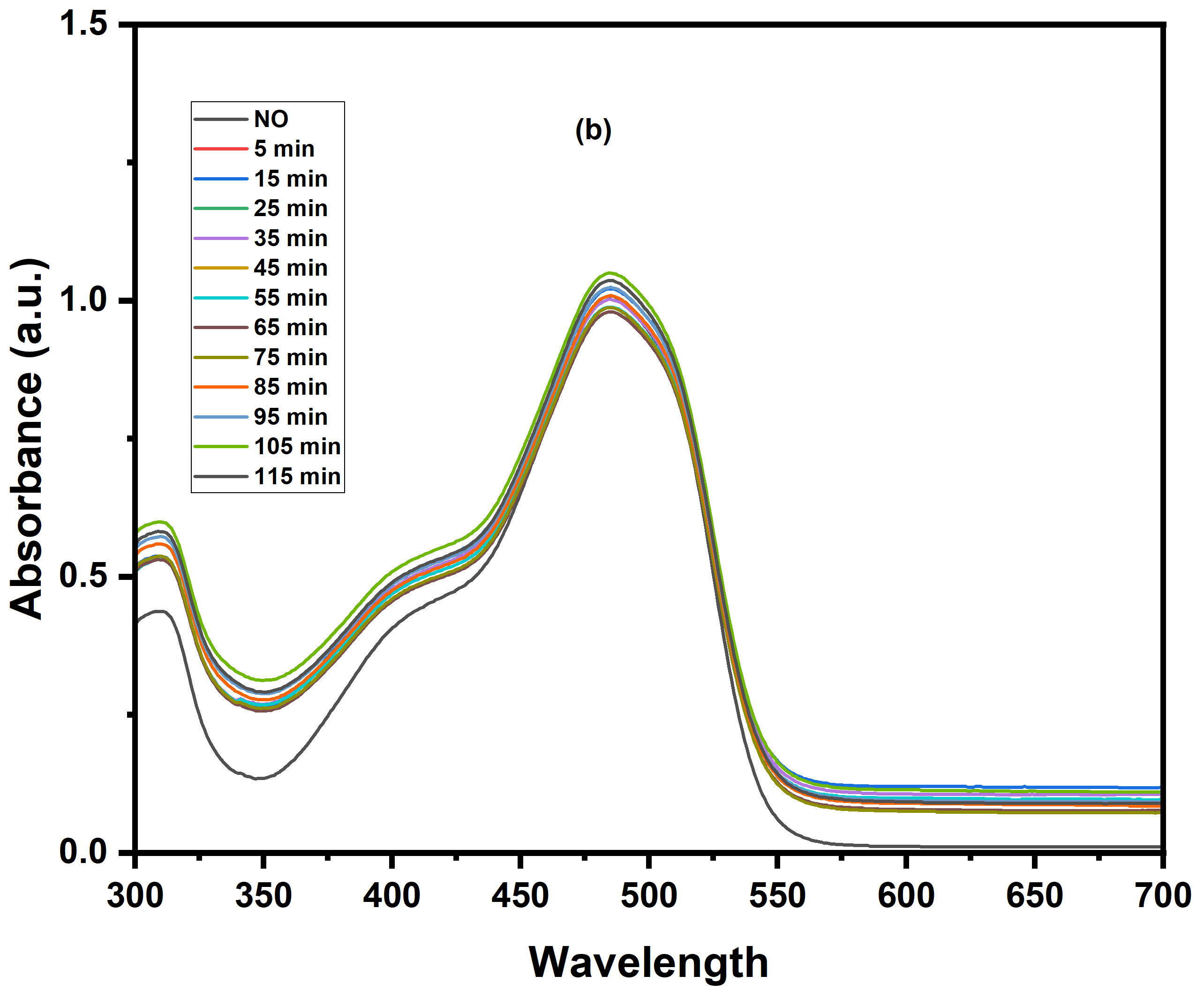

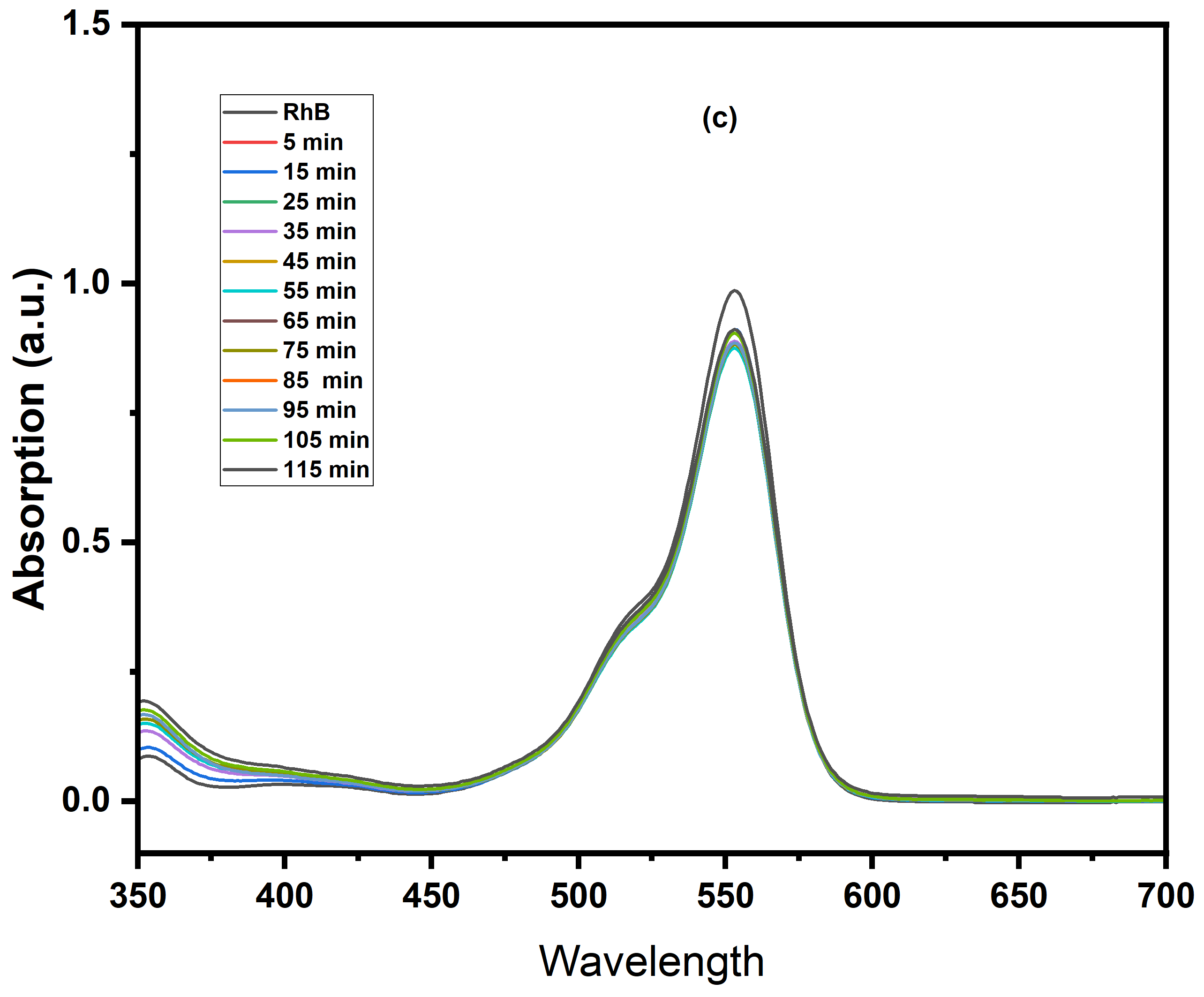


**Figure S2. UV-Vis spectra of different dye degradation MO (a), NO (b) and RhB (c) in presence of sunlight.**


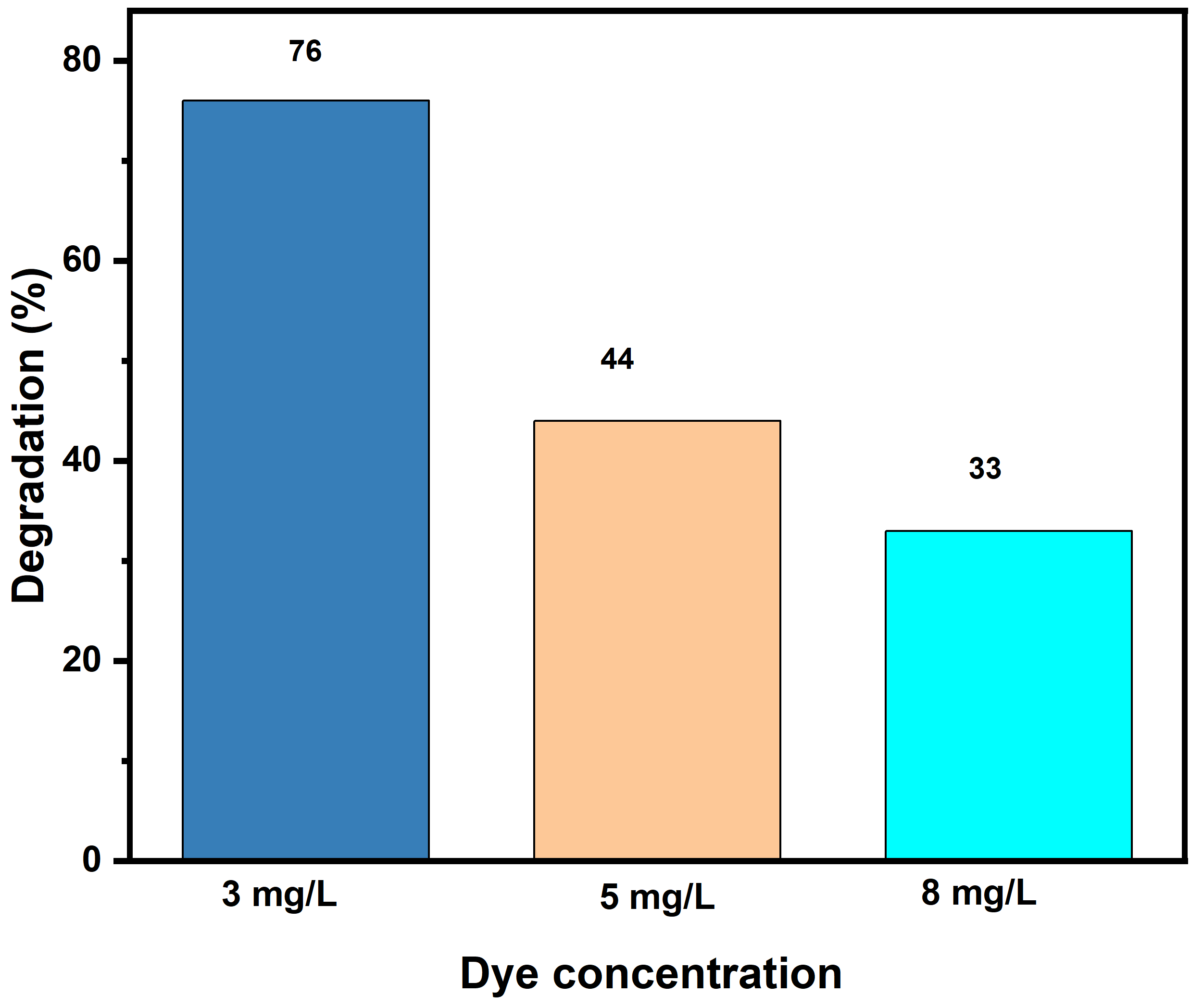


**Figure S3. Effect of initial dye concentration on the photodegradation of JC-La_2_CoO_4_ NPs**


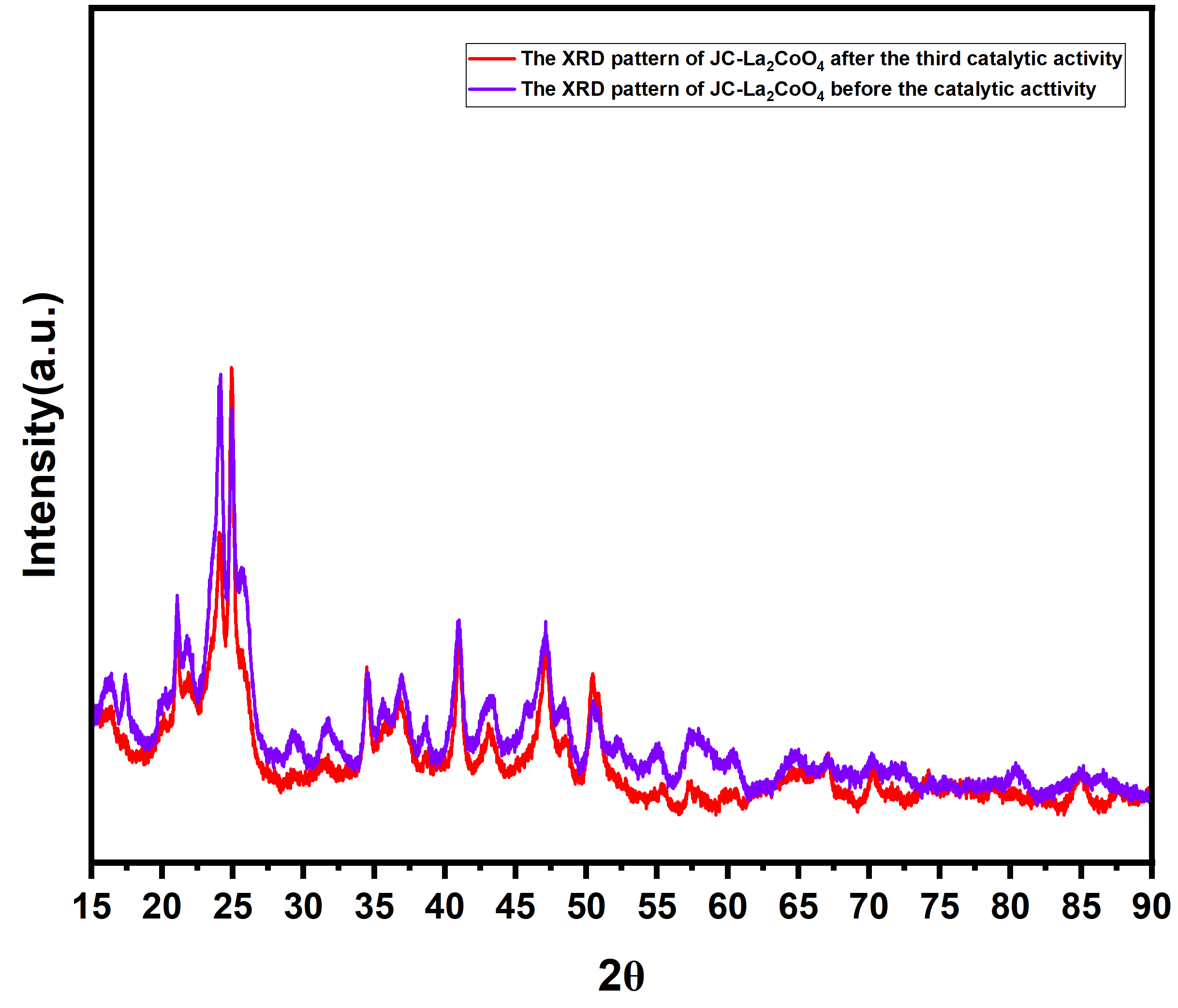


**Figure S4. The XRD pattern of JC-La_2_CoO_4_ before and after the third catalytic cycle.**

**Table S1**. Degradation rate constant (k) of MB, RhB, NO and MO summaries in the Table S1

| **S. No.** | **Dye** | **Rate constant (k)** |
| --- | --- | --- |
| 1 | MB | 56.73 × 10^-3^ |
| 2 | RhB | 0.000328 × 10^-3^ |
| 3 | NO | 0.000487 × 10^-3^ |
| 4 | MO | 0.000644 × 10^-3^ |

Degradation reaction Rate constant (k) was measured after 50% degradation of dyes with respect to time.

**Table S2: AC in phase and out phase**

| **Phase** | **Frequency (Hz)** | **χ_M_^'^T (cm^3^Kmol^-1^T)** | **T** |
| --- | --- | --- | --- |
| **In phase** | 50 | 0.00033 | 10 |
|  |  | 0.0015 | 100 |
|  |  | 0.0035 | 200 |
|  |  | 0.0054 | 300 |
|  | 250 | 0.00033 | 10 |
|  |  | 0.0016 | 100 |
|  |  | 0.003 | 200 |
|  |  | 0.0047 | 300 |
|  | 550 | 0.00033 | 10 |
|  |  | 0.0015 | 100 |
|  |  | 0.0029 | 200 |
|  |  | 0.0047 | 300 |
|  | **Frequency (Hz)** | **χ''M (cm^3^Kmol^-1^)** | **T** |
| **Out phase** | 50 | -0.000017 | 10 |
|  |  | -0.000015 | 100 |
|  |  | -0.0000083 | 200 |
|  |  | -0.000009 | 300 |
|  | 250 | -0.000069 | 10 |
|  |  | -0.000056 | 100 |
|  |  | -0.000046 | 200 |
|  |  | -0.000042 | 300 |
|  | 550 | -0.00015 | 10 |
|  |  | -0.00013 | 100 |
|  |  | -0.0001 | 200 |
|  |  | -0.00009 | 300 |
